# Development of strategies for genetic manipulation and fine‐tuning of a chloroplast retrograde signal 3′‐phosphoadenosine 5′‐phosphate

**DOI:** 10.1002/pld3.31

**Published:** 2018-01-09

**Authors:** Su Yin Phua, Wannarat Pornsiriwong, Kai Xun Chan, Gonzalo M. Estavillo, Barry J. Pogson

**Affiliations:** ^1^ ARC Centre of Excellence in Plant Energy Biology Research School of Biology The Australian National University Canberra ACT Australia; ^2^ Department of Biochemistry Faculty of Science Kasetsart University Bangkok Thailand; ^3^ CSIRO Agriculture & Food, Black Mountain Canberra ACT Australia

**Keywords:** 3′‐phosphoadenosine‐5′‐phosphate, drought, gene silencing, retrograde signaling, RNAi, SAL1

## Abstract

Homeostasis of metabolism and regulation of stress‐signaling pathways are important for plant growth. The metabolite 3′‐phosphoadenosine‐5′‐phosphate (PAP) plays dual roles as a chloroplast retrograde signal during drought and high light stress, as well as a toxic by‐product of secondary sulfur metabolism, and thus, its levels are regulated by the chloroplastic phosphatase, SAL1. Constitutive PAP accumulation in *sal1* mutants improves drought tolerance but can impair growth and alter rosette morphology. Therefore, it is of interest to derive strategies to enable controlled and targeted PAP manipulation that could enhance drought tolerance while minimizing the negative effects on plant growth. We systematically tested the potential and efficiency of multiple established transgenic manipulation tools in altering PAP levels in Arabidopsis. Dexamethasone (dex)‐inducible silencing of *SAL1* via hpRNAi [pOpOff:*SAL1*hpRNAi] yielded reduction in *SAL1* transcript and protein levels, yet failed to significantly induce PAP accumulation. Surprisingly, this was not due to insufficient silencing of the inducible system, as constitutive silencing using a strong promoter to drive hpRNAi and amiRNA targeting the *SAL1* transcript also failed to increase PAP content or induce a *sal1*‐like plant morphology despite significantly reducing the *SAL1* transcript levels. In contrast, using dex‐inducible expression of *SAL1* cDNA to complement an Arabidopsis *sal1* mutant successfully modulated PAP levels and restored rosette growth in a dosage‐dependent manner. Results from this inducible complementation system indicate that plants with intermediate PAP levels could have improved rosette growth without compromising its drought tolerance. Additionally, preliminary evidence suggests that *SAL1* cDNA driven by promoters of genes expressed specifically during early developmental stages such as *ABA‐Insensitive 3* (*ABI3*) could be another potential strategy for studying and optimizing PAP levels and drought tolerance while alleviating the negative impact of PAP on plant growth in *sal1*. Thus, we have identified ways that can allow future dissection into multiple aspects of stress and developmental regulation mediated by this chloroplast signal.

## 1 INTRODUCTION

Chloroplasts are one of the key organelles in plant cells, acting as the site of oxygenic photosynthesis while housing the biosynthesis of various important metabolites including amino acids, nucleotides, fatty acids, phytohormones, and sulfur assimilation (Bobik & Burch‐Smith, 2015). Effective communication between the chloroplast and the central regulator of a cell, the nucleus, is therefore necessary to coordinate cellular metabolism and growth. This interorganellar communication system can be generally categorized into anterograde (nucleus to organelle) and retrograde (organelles to nucleus) signaling (Woodson & Chory, 2008). A variety of chloroplast retrograde signals have been reported in the literature during the past decade including phosphoadenosines, carotenoids derivatives, heme, tetrapyrroles, and isoprenes (Chan, Phua, Crisp, Mcquinn, & Pogson, 2016). Most of these signals were discovered from analyses of mutants that have altered nuclear transcriptional responses to chloroplast perturbations. Although some of these mutants have acquired some form of stress tolerance (Estavillo et al., 2011; Mochizuki, Brusslan, Larkin, Nagatani, & Chory, 2001; Wilson et al., 2009; Xiao et al., 2012), they also present alterations in growth and hormonal signaling (Lemos et al., 2016; Robles et al., 2010; Rodríguez, Chételat, Majcherczyk, & Farmer, 2010; Rossel et al., 2006; Zhang et al., 2011).

The metabolite 3′‐phosphoadenosine‐5′‐phosphate (PAP), a by‐product of secondary sulfur metabolism, is a stress‐induced chloroplast retrograde signal in plants (Chan, Wirtz, Phua, Estavillo, & Pogson, 2013; Chan, Phua et al., 2016; Estavillo et al., 2011). PAP is present at very low levels in leaves of wild‐type *Arabidopsis thaliana* under standard growth conditions, presumably due to its degradation in the chloroplast by the phosphatase SAL1. Under oxidative stresses such as drought or high light, redox downregulation of SAL1 activity in the chloroplast (Chan, Mabbitt et al., 2016) enables PAP to accumulate and act as a stress signal in leaf tissues of *A*. *thaliana* (Estavillo et al., 2011). PAP can move from the chloroplast to the nucleus via the cytosol and it inhibits exoribonucleases (XRNs). This alters expression of many stress‐related genes, including abscisic acid (ABA)‐responsive ones, thus regulating drought stress signaling and responses (Estavillo et al., 2011). Accordingly, constitutive high PAP levels in the *sal1* mutant confers drought tolerance (Estavillo et al., 2011; Rossel et al., 2006; Wilson et al., 2009) and restores ABA signaling in ABA‐insensitive mutants (Pornsiriwong et al., 2017). However, these traits are offset by the fact that constitutively accumulated high PAP in *sal1* mutants also promotes pleiotropic changes in plant development (Kim & von Arnim, 2009; Robles et al., 2010; Wilson et al., 2009). For instance, Arabidopsis *sal1* mutants have shorter petioles, more rounded, serrated, and undulated leaves, slower developmental rate, and delayed flowering under some growth regimes.

3′‐Phosphoadenosine‐5′‐phosphate levels need to be tightly coordinated to ensure a balance between stress tolerance and correct plant development. Nonetheless, it is unknown if and how different PAP levels separately affect vegetative growth and plant drought tolerance. Indeed, to date no systematic analyses have been performed to dissect the primary (chloroplast signaling) and secondary (e.g., plant development) effects of retrograde signals in terms of dosage and temporal factors. Although PAP feeding on Arabidopsis plants has been described (Pornsiriwong et al., 2017), this approach is difficult, expensive, and subject to variability in chemical uptake efficiency. Thus, it is of interest to develop alternative genetic resources for controlled manipulation of PAP levels *in planta* to achieve stress tolerance while minimizing negative pleiotropic effects.

Hairpin RNA interference (hpRNAi) and artificial microRNA (amiRNA) are two gene‐silencing strategies successfully used in plants (de Felippes, Wang, & Weigel, 2012). Both techniques utilize double‐stranded RNA to generate small RNAs (sRNAs) via DICER‐LIKE (DCL) RNases. The sRNAs are subsequently loaded onto RNA‐induced silencing complexes (RISCs) as guides for targeting the repression of RNA that shares at least partial sequence complementation with them. For hpRNAi, a section of intended target sequence is cloned into a vector twice, each in opposite direction and separated by a linker of optimal length, enabling the formation of stem‐loop region upon transcription (Watson, Fusaro, Wang, & Waterhouse, 2005). Meanwhile, amiRNA modifies existing miRNA precursor sequences to match a portion of target sequence via overlapping PCRs (Schwab, Ossowski, Riester, Warthmann, & Weigel, 2006). Multiple sRNAs can be produced from the hpRNAi approach as opposed to the amiRNA approach that generates only a single sRNA (Ossowski, Schwab, & Weigel, 2008). Both strategies have been extensively used in plants; they can also be driven by inducible promoters, such as a dexamethasone (dex)‐inducible promoter (Wielopolska, Townley, Moore, Waterhouse, & Helliwell, 2005), which allows targeted manipulation of the gene of interest.

Here, we investigate the potential of genetically manipulating *SAL1* expression in wild‐type Arabidopsis, including the utilization of hpRNAi and amiRNA strategies under inducible and constitutive promoters, for adjusting the levels of the chloroplast retrograde signal PAP. Complementary strategies utilizing chemical‐inducible and developmental stage‐specific complementation of *sal1* were also tested. The efficiencies of these various strategies in enabling the alteration of PAP levels in Arabidopsis and the corresponding developmental effects are presented. Finally, drought tolerance was assessed on selected plants and insights into SAL1/PAP interaction with rosette growth and drought tolerance are discussed.

## 2 MATERIALS AND METHODS

### 2.1 Plasmid construction

Standard molecular biology techniques [detailed in (Sambrook & Russell, 2001)] were used in constructing plasmid vectors. All relevant DNA segments were amplified using Phusion polymerase (NEB, US) as per manufacturer's instructions. All intermediate and final PCR or cloning products were sequenced to confirm their identities.

The *SAL1*‐targeting hpRNAi constructs were generated by first using PCR primers SAL1‐RNAi‐F2 (5′‐AGAGGACTCAGGCGATCTAC‐3′) and SAL1‐RNAi‐R2 (5′‐CTTTTAGTGCCATCAATTGG‐3′) to amplify a 209‐bp product corresponding to nucleotides from 375 to 583 of the SAL1 CDS. The product was then inserted into the pCR8/GW/TOPO vector as per manufacturer's instructions, then inserted into either the pOpOff2(kan) vector (Wielopolska et al., 2005) or pAgrikola vector (Hilson et al., 2004) using Gateway LR reaction.

The *SAL1*‐targetting amiRNA constructs were designed using the Web MicroRNA Designer (WMD) platform (http://wmd3.weigelworld.org/cgi-bin/webapp.cgi) and constructed as detailed in Schwab et al. (2006) and Ossowski et al. (2008). The PCR primers used for generating the amiRNA fragment targeting the 5′ region of *SAL1* include amiRNA339‐F (5′‐GATGACTAAACTAACAACTGCTCTCTCTCTTTTGTATTCC‐3′), amiRNA339‐R (5′‐GAGAGCAGTTGTTAGTTTAGTCATCAAAGAGAATCAATGA‐3′), amiRNA*339‐F (5′‐GAGAACAGTTGTTAGATTAGTCTTCACAGGTCGTGATATG‐3′), amiRNA*339‐R (5′‐GAAGACTAATCTAACAACTGTTCTCTACATATATATTCCT‐3′) or 3′ region of *SAL1* include amiRNA1002‐F (5′‐GATAAACCGTAAGTATATAGCTCTCTCTCTTTTGTATTCC‐3′), amiRNA1002‐R (5′‐GAGAGCTATATACTTACGGTTTATCAAAGAGAATCAATGA‐3′), amiRNA*1002‐F (5′‐GAGAACTATATACTTTCGGTTTTTCACAGGTCGTGATATG‐3′), amiRNA*1002‐R (5′‐GAAAAACCGAAAGTATATAGTTCTCTACATATATATTCCT‐3′) in conjunction with Primer A (5′‐CTGCAAGGCGATTAAGTTGGGTAAC‐3′) and Primer B (5′‐GCGGATAACAATTTCACACAGGAAACAG‐3′).

The final PCR fragments were cloned into pCR8/GW/TOPO vector as per manufacturer's instructions and introduced into the pMDC32 vector (Curtis & Grossniklaus, 2003) for strong constitutive expression using Gateway LR reaction. The regions within *SAL1* targeted by the hpRNAi and amiRNA constructs are summarized in Figure S11.

For dexamethasone‐inducible complementation of *sal1*,* SAL1* cDNA was first amplified using SAL1cDNA_*Pac*I_F (5′‐GCTTAATTAAATGATGTCTATAAATTGTTTTC‐3′) and SAL1cDNA_R_*Avr*II_*Spe*I (5′‐AGACTAGTAGCCCTAGGTCAGAGAGCTGAAGCTTTCTC‐3′). The PCR product was cloned into pMDC123 vector (Curtis & Grossniklaus, 2003) using *Pac*I and *Spe*I sites. NOS terminator was amplified from pMDC32 (Curtis & Grossniklaus, 2003) using NOST_F_*Avr*II (5′‐TCCCCTAGGATCGTTCAAACATTTGGC‐3′) and NOST_R_*Spe*I (5′‐AGACTAGTAATTCAGTAACATAGATGAC‐3′) and then inserted downstream of *SAL1* cDNA using *Avr*II and *Spe*I sites. The *SAL1*nosT fragment was then PCR‐amplified and cloned into pCR8/GW/TOPO vector as per manufacturer's instructions and then introduced into the pOpON(hyg) vector [a variation of pOpOff2(hyg), obtained from Dr. Chris Helliwell from CSIRO Plant Industry, Australia] using Gateway LR reaction.

For early developmental stage‐specific complementation of *sal1*, the *SAL1*nosT fragment generated earlier was inserted downstream of the Gateway‐compatible cassette via restriction enzyme digestion and ligation in the pMDC123 vector (Curtis & Grossniklaus, 2003) using *Pac*I and *Spe*I sites. Meanwhile, the promoter region of *ABI3* (*ABA‐Insensitive 3*), *TZF6* (*Tandem CCCH Zinc Finger Protein 6*; also known as PEI1) and *LEC1* (*Leafy Cotyledon 1*) were amplified using ABI3pro_F (5′‐CACCTGGTGATCGGAAAATCCGAGG‐3′) and ABI3pro_R (5′‐AAACTAGATTGGTGGAGAGAGAAAA‐3′), PEI1pro_F (5′‐CACCCCTTGTAAACTGGCATAAATTCTGA‐3′) and PEI1pro_R (5′‐TTTCCTTGCAATGATCTAAAGAGTT‐3′), or LEC1pro_F (5′‐CACCCTTTATGGGCTGCTTGTTC‐3′) and LEC1pro_R (5′‐TGTTTCTCTGCCGTCTTTT‐3′), respectively. Forward primer for amplifying all three promoters has “CACC” added at the 5′ end as per manufacturer's instruction for directional cloning into the pENTR™ Directional TOPO^®^ vector (Life Technologies, USA). The promoters were cloned into the entry vector as per manufacturer's instructions and then introduced into the pMDC123‐*SAL1*nosT vector (no pro:*SAL1*) using Gateway LR reaction (Figure 6a). Col‐0 transformed with empty vector (containing BASTA‐resistant gene only) was used as control.

### 2.2 Plant transformation and growth

*Arabidopsis thaliana* wild‐type Col‐0 or *sal1* null mutant allele *fry1‐6*, denotes as *sal1‐*6 in this manuscript, (a T‐DNA mutant from ABRC–SALK_020882) in Col‐0 ecotype background was transformed using *Agrobacterium*‐mediated floral dipping method (Clough & Bent, 1998; Zhang, Henriques, Lin, Niu, & Chua, 2006). Seeds were stratified at 4°C for 2‐3 days before growth under standard conditions of 100–150 μmol photons m^−2^ s^−1^, 21–23°C at 16‐hr photoperiod, either on Seedling Raising Mix (Debco, Australia) supplemented with 3 g/L Osmocote Exact Mini (Scotts, Australia) or on agar‐solidified Murashige and Skoog (MS) media (Austratec, Australia). Seeds were sterilized with 1% (v/v) HCl/bleach for 4 h before plating on media. Transgenic plants were selected by spraying soil‐germinated seedlings with 50 mg/L BASTA (glufosinate‐ammonium salt; Sigma‐Aldrich, USA) herbicide or by growing on MS supplemented with either 50 mg/L kanamycin (kanamycin sulfate; Sigma‐Aldrich) or 100 mg/L hygromycin B (Life Technologies/ThermoFisher Scientific, USA). For dexamethasone‐inducible *SAL1* silencing or expression, plants were treated with 20 μM of water‐soluble dexamethasone (Sigma‐Aldrich) through soil drenching or media, or supplemented with 0.02% (v/v) Silwet L‐77 when painted or sprayed directly on leaves/rosettes. For all drought stress experiments, plant survival was assessed from chlorophyll fluorescence measurements (maximum efficiency of photosystem II, Fv/Fm) according to method described by Woo, Badger, and Pogson (2008).

### 2.3 RNA isolation and *SAL1* transcript quantification

Total RNA was extracted using Sigma‐Aldrich Spectrum™ Plant Total RNA Kit, with On‐Column DNase digestion step performed as per manufacturer's instructions. First‐strand cDNA synthesis was performed using SuperScript^®^ III Reverse Transcriptase (Invitrogen) with Oligo(dT) primer and 1 μg total RNA (quantified using ND‐1000 spectrophotometer [NanoDrop Technologies, USA]) following guidelines from the manufacturer. The resulting cDNA was diluted five times and 1 μl of the diluted cDNA was mixed with 5 μl Roche LightCycler 480 SYBR Green I Master Mix and 0.4 μM of each primer (final reaction volume of 10 μl) for each qPCR in a 384‐well plate. The qRT‐PCR was performed on Roche LightCycler 480 with three technical replicates per sample, using the amplification conditions of: 10 mins at 95°C (ramp rate of 4.8°C/s), 50 cycles of 10 s at 95°C (4.8°C/s), 30 s at 62°C (2.5°C/s), 30 s at 60°C (2.5°C/s), before introducing a final melting temperature of 95°C for 30 s at a ramp rate of 2.5°C per second. *Glyceraldehyde‐3‐phosphate dehydrogenase C2* (*GAPC2*, AT1G13440) was used as reference control. The Roche LightCycler 480 Software (Relative Quantification Fit Point method) was used to analyze the qRT‐PCR results, and the relative transcript abundance was calculated using the formula: Target Eff^Ct(Wt–target)^/Reference Eff^Ct(Wt–target)^ (Pfaffl, 2001). Primer sequences for qRT‐PCR are as follows: SAL1_LP2 (5′‐CTGAAGGTGGTCCAAATGGT‐3′), SAL1_RP2 (5′‐TGATCTCCCCTCAGAAATCC‐3′), GAPC2_3′F (5′‐ACAGTTCTCGTGTCGTTGACC‐3′), and GAPC2_3′R (5′‐ACCACACACAAACTCTCGCC‐3′).

### 2.4 SAL1 protein analyses

Total protein from tissues was extracted in cold acetone containing 10% (w/v) tricarboxylic acid and 0.07% (w/v) dithiothreitol (DTT). Pellet was washed twice in cold acetone containing 0.07% DTT before drying and resuspending in urea buffer (9 M urea, 4% [w/v] CHAPS, 1% [w/v] DTT, 35 mM Tris base). Total protein extracted was quantified using Bradford assay (dye from Bio‐Rad, USA), and different concentrations of bovine serum albumin (BSA) (Sigma‐Aldrich) solubilized in urea buffer were used as standards. Western blots were performed as previously described (Wilson et al., 2009). In brief, 5 μg of leaf total protein extract and 5 ng of recombinant SAL1 (rSAL1) used as a positive antibody specificity control were resolved on 4%–12% (w/v) SDS‐PAGE (NuPAGE; Invitrogen), electrotransferred to a PVDF membrane and probed with a 1:1,000 dilution of polyclonal antibodies raised against rSAL1 (Wilson et al., 2009) for 10 min using the SNAP i.d. system (Millipore, USA). After three washes with PBS, the blot was incubated with 1:10,000 dilution of HRP‐conjugated goat anti‐rabbit IgG for 10 min, washed three times, and developed using the Amersham ECL Prime Western Blotting Detection Reagent (GE Healthcare, UK). The chemiluminescence was visualized under FUSION‐SL Chemiluminescence System (Vilber Lourmat, France).

### 2.5 Quantification of phosphoadenosines

Total adenosines were extracted with 0.1 M HCl before derivatization with chloroacetaldehyde and finally quantified fluorometrically upon HPLC fractionation as previously described (Estavillo et al., 2011). Corresponding HPLC peak area was integrated and converted to pmol units using standard curves of 1, 5, and 10 pmol standards for PAP quantification (Burstenbinder, Rzewuski, Wirtz, Hell, & Sauter, 2007).

### 2.6 Histochemical localization of β‐glucuronidase activity

β‐glucuronidase staining was performed based on the method described in Jefferson et al. (1987) and Millar and Gubler (2005). In brief, leaf tissues or whole seedlings were harvested and soaked individually in each well of a 24‐well plate containing 500 μl to 1 ml of GUS staining solution [100 mM sodium phosphate buffer at pH 7.0, 0.1% (v/v) Triton X‐100, 2 mM potassium ferricyanide, 2 mM potassium ferrocyanide, and 1 mg/ml 5‐bromo‐4‐chloro‐3‐indolyl‐β‐d‐glucuronide] per well. Samples were vacuum‐infiltrated for 5–10 min before incubating at 37°C for overnight. Samples were incubated at room temperature in a series of 500 μl to 1 ml ethanol (20%, 50% and 70% [v/v]) per well for 1–2 hr each, and cleared samples were photographed with Lumix DMCFZ5 camera (Panasonic, Japan).

### 2.7 Plant imaging

Imaging of Arabidopsis was performed using the Scanalyzer (LemnaTec, Germany), an automatic image capturing and processing system that can measure multiple parameters of Arabidopsis plants, including leaf color, rosette area in units of pixels, and rosette compactness in fraction form. Rosette area quantified was expressed in pixels and converted into cm^2^ using the average conversion coefficient of 1402.25 (see Figure S12 for the calibration curve for the conversion).

### 2.8 Statistical analyses

The two‐sample Student *t* test (assuming equal variance) was performed in Microsoft Excel 2016 when comparing two sample groups of interest. Multiple‐way analysis of variance (ANOVA) followed by Tukey's honestly significant difference (HSD) post hoc test was performed using R software version 3.3.2 (http://www.rproject.org/) when the analyses involved more than one independent variable. No statistics was performed when no replicate is available. Boxplots were plotted using default settings in Microsoft Excel 2016.

## 3 RESULTS

### 3.1 *SAL1* transcript repression by dexamethasone(dex)‐inducible *SAL1*hpRNAi could not induce PAP accumulation

**Figure 1:**
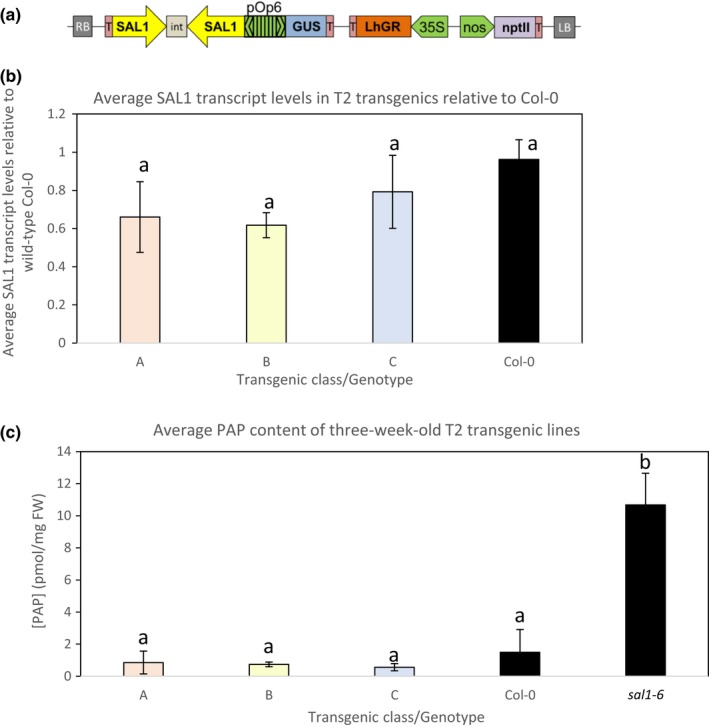
Dexamethasone‐inducible SAL1 silencing using hpRNAi pOpOff system. (a) Schematic diagram of the dex‐inducible pOpOff2(kan)‐SAL1hpRNAi plasmid vector. RB, right border; T, terminator; int, intron; nptII, kanamycin‐resistant gene; LB, left border. Three‐week‐old seedlings germinated and grown on MS supplemented with 20 μM dex were harvested for (b) SAL1 transcript quantification relative to the wild‐type Col‐0 control via qRT‐PCR and (c) 3′‐phosphoadenosine‐5′‐phosphate quantification using HPLC. At least 10 seedlings per transgenic line were pooled for each quantification. Significant differences (ANOVA, *p* < .05) are denoted by a, b. *n* = 3–4; error bars = *SD*

The pOpOff2(kan) vector system (Wielopolska et al., 2005) was utilized to chemically control the expression levels of *SAL1*. This vector system contains a dex‐inducible bidirectional promoter driving the expression of both the GUS reporter gene and the Gateway‐compatible cassettes for hpRNAi (Figure 1a). Transgenic plants carrying the pOpOff‐*SAL1*hpRNAi vector were isolated by kanamycin selection. To induce gene expression, whole or parts of leaves from T1 plants were treated with 20 μM dex and GUS staining was performed to check for reporter gene expression (Figure S1a). Strong GUS staining was only observed in leaf sections or whole leaf where dex was applied, and the effect persisted for up to 2 days post‐treatment.

Subsequently, leaves of more than twenty T2 transgenic lines were treated with 20 μM dex and GUS‐stained to verify the responsiveness of gene expression to dex treatment. Five progenies per line were tested to account for any possible technical and biological variables. Two representative staining results are shown for each line (Figures S1b and S2a). Most of the T2 individuals showed positive GUS staining, albeit with different intensities. We investigated if the GUS staining intensities for each line is correlated with their corresponding *SAL1*‐silencing efficiencies by quantifying *SAL1* transcript levels and PAP levels (Figure 1b,c; Figure S2b,c). Despite the presence of GUS staining and reduction in *SAL1* transcript levels, there was no significant PAP accumulation. Further, although strong GUS staining was observed in some lines, the maximum reduction in *SAL1* transcript achieved using this dex‐inducible *SAL1*‐silencing system was only at most 70%. Interestingly, *SAL1* transcript levels in *sal1* were higher than wild type*,* most likely caused by feedback regulation due to absence of functional SAL1 protein (data not shown). The maximum average of PAP accumulation detected in the transgenic lines was still within the range of PAP levels in the wild‐type Col‐0, which was approximately 10 times lower than that in *sal1*.

We then examined the *SAL1* transcript levels in T3 homozygous lines to assess heritability of this trait. We found that the extent of transcript reduction upon dex treatment was either comparable or lesser than that of the parents (Figure S1c). Nonetheless, a reduction in SAL1 protein levels was still detected even though the PAP levels remained comparable to that of wild‐type Col‐0 (Figure S1d). These results suggest that the *SAL1* transcript reduction and the associated decrease in protein levels achieved using this dex‐inducible system may not be sufficient to significantly induce PAP accumulation in the transgenic lines relative to wild‐type Col‐0. To test this hypothesis, we investigated whether *sal1* phenotypes can be recapitulated using the same hpRNAi construct driven by a strong constitutive promoter.

### 3.2 *SAL1*hpRNAi under strong constitutive promoter also could not induce PAP or alter rosette growth

**Figure 2:**
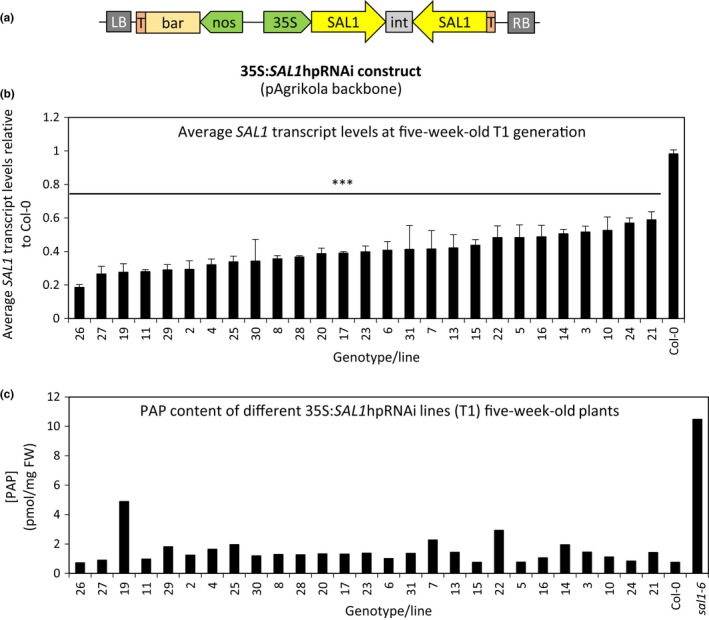
Generating and testing the potential of *SAL1*‐silencing efficiency using hpRNAi under CaMV35S strong constitutive promoter. (a) Schematic diagram of the 35S:*SAL1*hpRNAi (pAgrikola as backbone) plasmid vector. RB, right border; T, terminator; int, intron; bar, BASTA‐resistant gene; LB, left border. Leaves of five‐week‐old T1 transformants were harvested for (b) *SAL1* transcript quantification [*n* = 3 technical replicates, error bars = *SD*, significant differences = ANOVA, post hoc test relative to Col‐0: ***p < .001] and (c) 3′‐phosphoadenosine‐5′‐phosphate quantification [*n* = 1]

The pAgrikola vector (Hilson et al., 2004) contains a strong constitutive promoter that drives the expression of the hpRNAi cassette, identical to that of pOpOff2(kan) vector system. Hence, the pAgrikola vector was used for recreating the *SAL1* hpRNAi to test its maximal capacity in silencing the endogenous *SAL1* in Arabidopsis (Figure 2a).

To avoid confounding effects from poorer silencing efficiency in subsequent generations, which was observed in the dex‐inducible SAL1‐hpRNAi system, we studied the first generation (T1) transformants carrying the 35S:*SAL1*hpRNAi construct. Leaf tissues of the isolated BASTA‐resistant T1 transgenic lines were harvested for *SAL1* transcript level and PAP level quantification (Figure 2b,c). Upon screening about 30 lines, the minimum reduction in *SAL1* transcript levels of 35S:*SAL1*hpRNAi transgenic lines relative to wild type is 40% while a maximum reduction of slightly more than 80% in *SAL1* transcript levels was achieved under the strong constitutive promoter. However, the PAP levels remained comparable to that of wild‐type Col‐0 (Figure 2c) and were not directly correlative to their corresponding *SAL1* transcript levels (Figure 2b). This wild‐type‐like PAP levels remained in the subsequent T2 generation when leaf tissues of a few representative lines were sampled (Figure S3b). Additionally, rosette morphology of all transformants remained similar to that of wild‐type Col‐0 (Figure S3a).

Given the inability of the hpRNAi cassette to sufficiently silence *SAL1* expression such that PAP levels and leaf morphology could be altered, we reasoned that an alternative transcript silencing method, artificial miRNA (amiRNA) targeting different regions of *SAL1* compared to that targeted by the hpRNAi, should be tested.

### 3.3 No PAP accumulation nor altered rosette growth in *2X35S:SAL1*amiRNA transgenics

**Figure 3:**
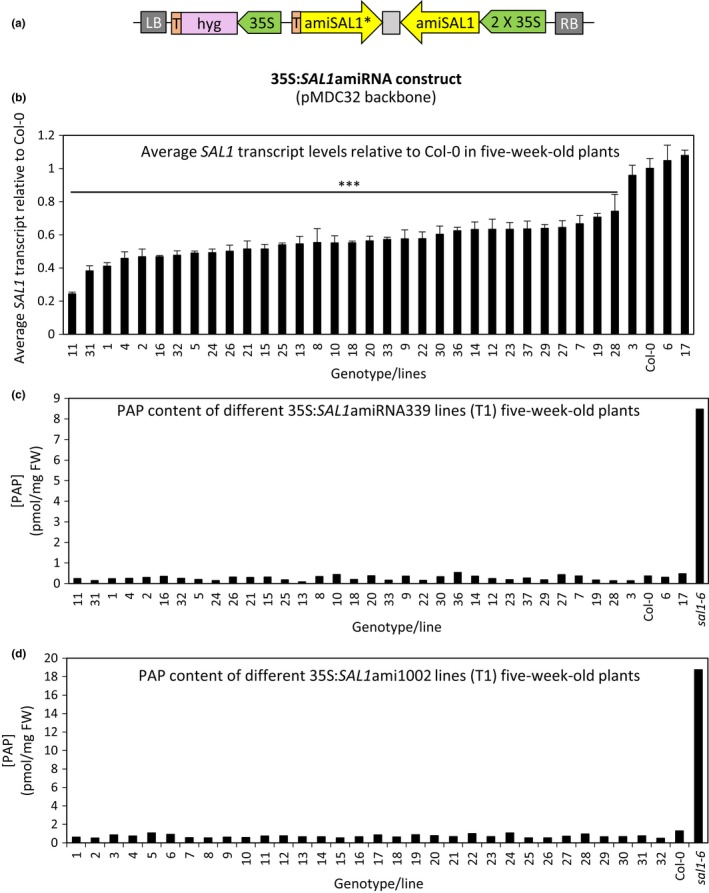
*SAL1*‐silencing efficiency using amiRNA under CaMV35S strong constitutive promoter. (a) Schematic diagram of the 35S:*SAL1*amiRNA (pMDC32 as backbone) plasmid vector. RB, right border; T, terminator; int, intron; hyg, hygromycin‐resistant gene; LB, left border. Two variations of SAL1amiRNA targeting 5′ and 3′ regions of *SAL1*, respectively, were tested. Leaves of five‐week‐old T1 transformants with amiRNA targeting the 5′ region of *SAL1* (35S:*SAL1*ami339) were harvested for (b) *SAL1* transcript quantification [*n* = 3 technical replicates, error bars = *SD*, significant differences = ANOVA, post hoc test relative to Col‐0: ***p < .001] and (c) 3′‐phosphoadenosine‐5′‐phosphate (PAP) quantification [*n* = 1]. (d) PAP quantification of five‐week‐old T1 transformants with amiRNA targeting the 3′ region of *SAL1* (35S:*SAL1*ami1002) [*n* = 1]

Two different amiRNA constructs, targeting the 5′ and 3′ ends of *SAL1*, respectively*,* were amplified and inserted under the double CaMV35S strong constitutive promoter in pMDC32 (Curtis & Grossniklaus, 2003). They were named according to the bp region of *SAL1*cDNA targeted: amiRNA339 and amiRNA1002 for the 5′‐targeting and the 3′‐targeting constructs, respectively (Figure 3a).

More than 30 transgenic lines for each of the two different 35S:*SAL1*amiRNA constructs were isolated based on hygromycin resistance selection. *SAL1* transcript levels and PAP levels were quantified as before from leaf tissues of T1 35S:*SAL1*amiRNA339 transgenics (Figure 3b,c) while only PAP levels were quantified for 35S: *SAL1*amiRNA1002 transgenics (Figure 3d). Interestingly, the *SAL1* transcript repression by amiRNA appears less efficient than the hpRNAi tested as the maximum *SAL1* transcript reduction achieved is less than 80% (Figure 3b) while a few transgenic lines even showed comparable transcript levels to that of wild‐type Col‐0. Not surprisingly, PAP levels quantified for the transgenic lines (both for 35S:*SAL1*amiRNA339 and 1002) were comparable to wild type (Figure 3c,d, respectively) and most of the rosette morphology (leaf shape and rosette compactness) remained comparable to that of Col‐0 (Figure S4). Therefore, collectively we found that four different silencing strategies failed to adequately decrease *SAL1* expression to enable PAP accumulation and alter rosette morphology (Figure S5).

### 3.4 Dex‐inducible *SAL1* complementation enables PAP manipulations and rosette growth alterations

**Figure 4:**
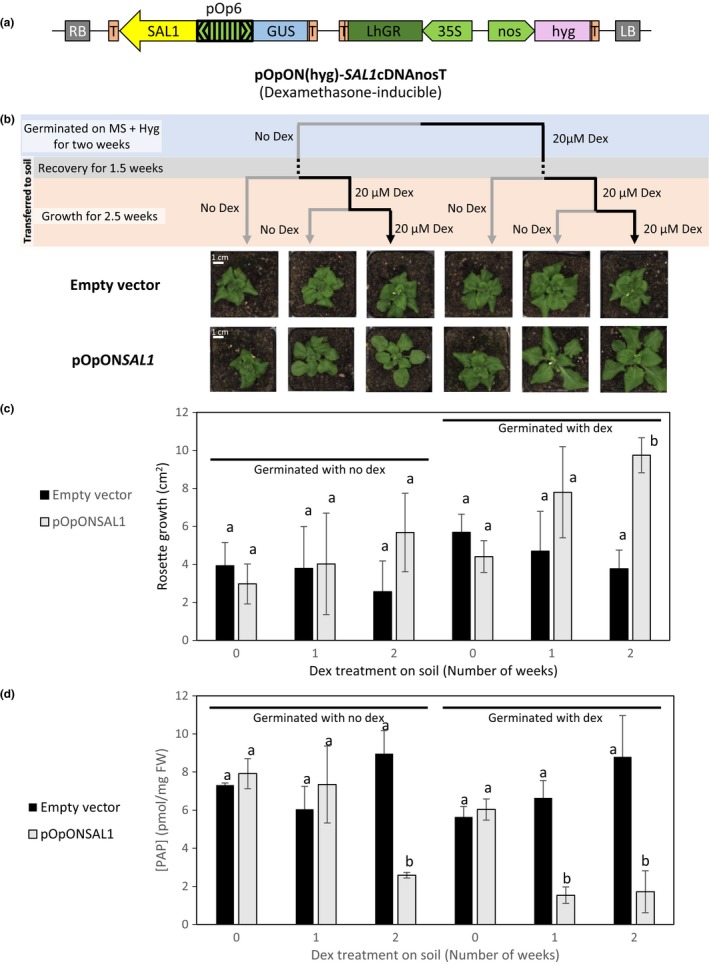
Inducible‐*SAL1* complementation of *sal1*‐6 using pOpON system for PAP manipulation. (a) Schematic diagram of the pOpON‐*SAL1* [pOpON(hyg) as backbone] plasmid vector. RB, right border; T, terminator; hyg, hygromycin‐resistant gene; LB, left border. (b) T2 transgenic lines of pOpON empty vector and pOpON*SAL1* were germinated under two different conditions: MS with hygromycin only and MS with hygromycin and 20 μM dex. After 2 weeks of growth on MS (blue phase), the plants were transferred to soil and let to adapt to growth on soil for 1.5 weeks (gray phase) before further dex treatment during growth on soil (orange phase). Representative images for both empty vector control and pOpON‐*SAL1* transgenic lines at the end of the different dex treatment regimes are shown; scale bars indicate 1 cm applicable to all photographs. (c) The corresponding average rosette growth quantified during the 2‐week dex treatment on soil is shown. (d) 3′‐phosphoadenosine‐5′‐phosphate quantification of the T2 transgenics at the end of the different dex treatment regimes. Error bars indicate standard deviation while statistical differences are denoted by different letters (a, b) above each bar based on two‐way ANOVAs

Successful *SAL1* complementation using strong constitutive or its endogenous promoter in different *sal1* mutant alleles has been demonstrated multiple times (Kim & von Arnim, 2009; Rodríguez et al., 2010; Wilson et al., 2009). We tested if a chemical‐inducible *SAL1* complementation system in a *sal1* mutant could be a successful approach to manipulate PAP levels in Arabidopsis. Full‐length *SAL1* cDNA was inserted into the pOpON(hyg) vector, a variation of pOpOff(hyg) (Wielopolska et al., 2005), where gene expression is also driven by the bidirectional dex‐inducible promoter (Figure 4a).

Both the pOpON empty vector and the pOpON‐*SAL1* vector were transformed into the *sal1* mutant allele, *sal1‐6*. The T2 transformants isolated based on hygromycin resistance were subjected to different regimes of dex treatment as illustrated in Figure 4b. When germinated on MS agar under hygromycin selection, transformants were subjected to either control or 20 μM dex treatment for 2 weeks. Once established, they were transplanted onto soil and allowed to recover for one and a half weeks before being subjected to either blank treatment, or 1 or 2 weeks of dex treatments. As expected, no significant growth difference was observed within transformants carrying the empty vector under the different dex treatment regimes. Significantly, faster rosette growth correlating to the length of dex treatment can be observed for the pOpON‐*SAL1* transformants at the end of the treatments both visually (Figure 4b) or when rosette growth was quantified by image analysis (Figure 4c; Figure S7). Transformants treated with dex throughout the experiment showed significantly higher rosette growth rate relative to their non‐treated counterparts as well as to the empty vector control.

Leaf tissues of the respective transformants were harvested at the end of all treatment regimes for PAP quantification. As expected, PAP levels remained high in the pOpON empty vector controls regardless of the dex treatment regimes (Figure 4d). Significantly, we observed a correlation between the rosette growth, PAP levels in the pOpON‐*SAL1* transformants, and the length of dex treatment throughout plant growth: Longer dex treatments correlated with lower PAP levels and better rosette growth. In agreement with the inducible nature of the *SAL1* expression, at least 2 weeks of constant dex treatment closer to the tissue harvesting time point was crucial for pOpON‐*SAL1* transformants to show lower PAP levels relative to the controls, whereas no dex treatment on soil or only 1 week of dex treatment on soil with no dex treatment during early development resulted in *sal1*‐like high PAP levels.

**Figure 5:**
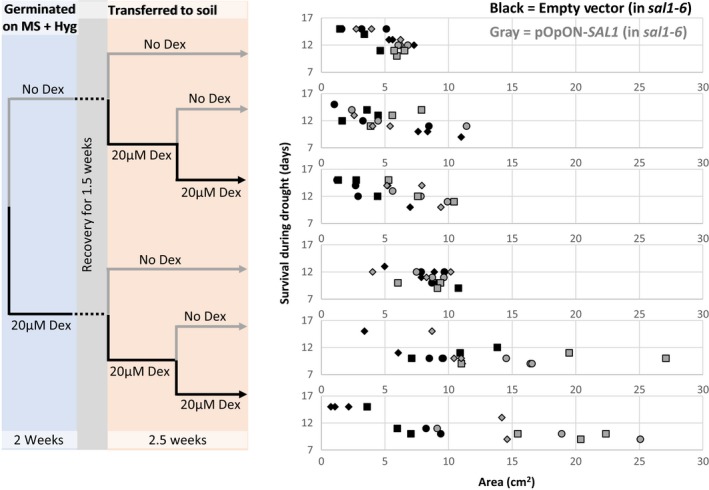
Comparing the days of survival during drought between *sal1*‐6 transformed with the empty vector control (black) and *sal1*‐6 complemented with pOpON‐*SAL1* (gray) upon different dex treatment regimes, relative to rosette size. Independent transgenic lines are depicted as different shapes

We investigated whether the dex‐inducible complementation of growth in the pOpON‐*SAL1* transformants negatively impacted drought tolerance. Interestingly, the drought tolerance, as indicated by the number of days of survival during drought, remained largely comparable between the pOpON empty vector controls and pOpON‐*SAL1* lines despite the improved rosette growth in pOpON‐*SAL1* (Figure 5), although the drought tolerance was not as prominent under the “constitutive” dex treatment.

### 3.5 *sal1* complemented with ABI3:*SAL1* showed intermediate PAP levels and improved rosette growth

**Figure 6:**
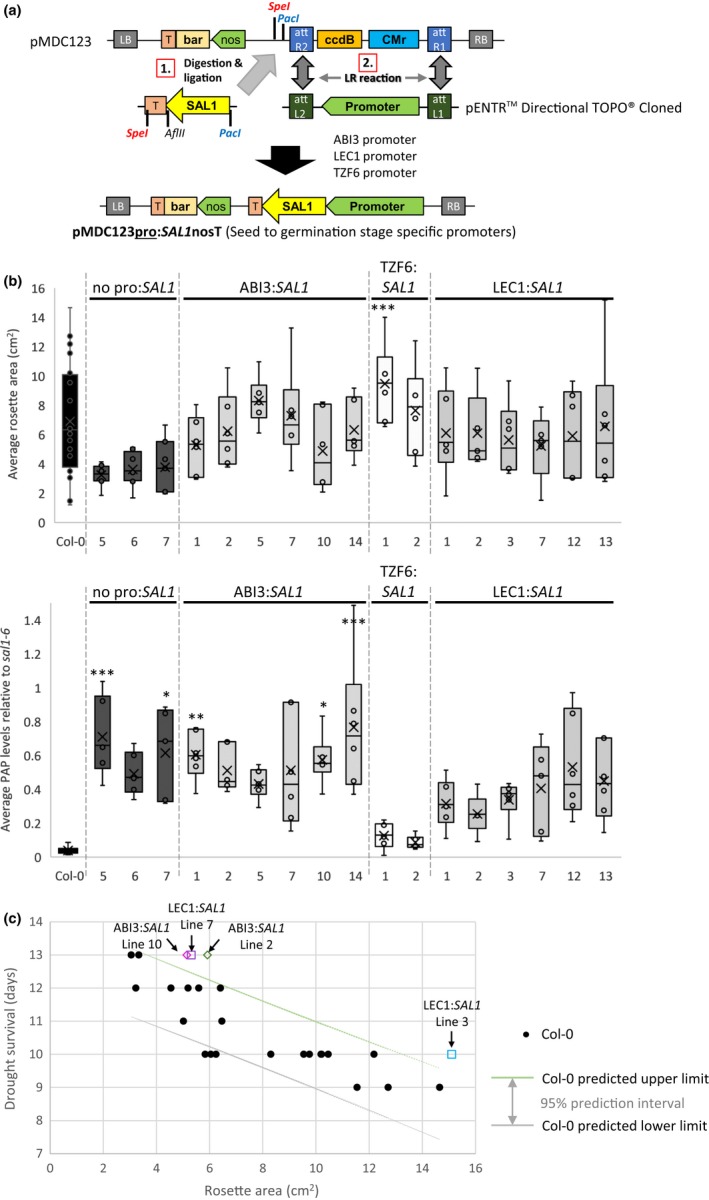
The effect of early developmental stage‐specific *SAL1* complementation of *sal1*‐6 using *ABI3*,* TZF6*, and *LEC1* promoter on rosette growth and 3′‐phosphoadenosine‐5′‐phosphate (PAP) levels. (a) Schematic diagram illustrating the cloning process of *SAL1* cDNA under different promoter using pMDC123 vector as the final destination vector. RB, right border; T, terminator; bar, BASTA‐resistant gene; LB, left border; att, compatible recombination attachment sites. (b) Rosette area (top) and PAP levels (bottom) of T2 transgenic lines at 4.5 weeks old were quantified; significant differences = ANOVA, post hoc test relative to Col‐0: *** p < 0.001, ** p < 0.01, * p < 0.05, *n* ≥ 5. (c) The correlation between the days of survival during drought and their corresponding rosette area for Col‐0 (BASTA‐resistant) control. ABI3:*SAL1* and LEC1:*SAL1* transgenic individuals showing better drought survival for a given ro sette size are highlighted; different shape denotes different transgenics; different colors denote different independent lines

We sought to further investigate the capacity for temporal control of *SAL1* expression in complementing *sal1* for balancing growth and drought tolerance at adult stage, utilizing appropriate endogenous promoters without relying on chemical treatment. As dex treatment on the dex‐inducible *SAL1* complementation lines from germination onwards was not able to fully restore *sal1* growth as the published constitutive complementation of *sal1* did, we hypothesized that the presence of functional SAL1 during embryogenesis to germination stages could be a more effective approach to improve rosette growth in a *sal1* mutant background. Hence, we used three different promoters with well‐characterized embryo‐ and seed‐specific expression to drive the expression of *SAL1* cDNA: *ABI3* [expressed throughout seed development and transiently after germination in organs of embryonic origin (Brady, Sarkar, Bonetta, & McCourt, 2003; Parcy et al., 1994)], *TZF6* [expressed in developing embryos only until just before desiccation (Bogamuwa & Jang, 2013; Li & Thomas, 1998)] and *LEC1* [expressed in early developing siliques of preglobular to heart stage up to heart and curled cotyledon stage, and not in maturing embryo stage (Lotan et al., 1998)]. The cloning strategies involved are summarized in Figure 6a.

Five (#1, 2, 3, 4, and 8) of the ten characterized T1 lines, transformed with control vector (*SAL1* cDNA with no promoter), showed complemented rosette phenotype and PAP levels (Figure S8), suggesting that the *SAL1*cDNA might have been expressed (most likely by the activation of a nearby upstream promoter). Indeed, when GUS reporter gene (containing ATG) without promoter was randomly inserted into the genome of Arabidopsis, 54% of the transformed plants showed the GUS expression (Kertbundit, De Greve, Deboeck, Van Montagu, & Hernalsteens, 1991), which is comparable to our observation here. Hence, only lines #5, 6 and 7 of no pro:*SAL1*, which retained the *sal1*‐like rosette size and PAP levels, were used in the subsequent generation as negative controls (Figure 6b; Figure S9). Different ABI3:*SAL1* transgenic lines showed a range of rosette size and PAP levels complementation relative to *sal1* at T1 generation. However, this was not the case for most lines carrying TZF6:*SAL1* or LEC1:*SAL1* lines, which displayed almost complete complementation with wild‐type‐like rosette morphology and PAP levels (Figure S8).

Phenotypes similar to those in T1 plants were observed in the subsequent T2 generation. A range of rosette sizes and PAP levels were present in ABI3:*SAL1* lines and to a certain extent in LEC1:*SAL1* but not in TZF6:*SAL1* lines (Figure S9, top and bottom, respectively). Selected ABI3:*SAL1* and LEC1:*SAL1* lines with higher‐than‐wild‐type PAP levels and increased rosette size relative to the negative control (no pro:*SAL1*) are shown in Figure 6b together with the controls and both lines of TZF6:*SAL1*. The range of variation in rosette size within each line was fairly consistent across the different independent transgenic lines despite having varying maximum and minimum values. On the other hand, the variation in PAP levels within a transgenic line is relatively small for majority of the lines. The minimum PAP levels of the selected ABI3:*SAL1* lines in Figure 6b are comparable to that of no pro:*SAL1* lines, whereas all TZF6:*SAL1* and the majority of LEC1:*SAL1* minimum PAP levels are more similar to that of Col‐0. Interestingly, two transgenic lines (lines #1 and 2 of ABI3:*SAL1*) consistently showed overall PAP levels comparable to that of *sal1* with an overall improved rosette size in both T1 and T2 generations. This suggests that rosette size could be uncoupled from PAP levels, albeit in a transgenic event‐dependent manner.

Therefore, we sought to establish whether drought tolerance in these lines could similarly be uncoupled from rosette size and PAP. We performed drought assays and rosette size quantification on at least 20 Col‐0 plants with a range of rosette sizes. This enabled us to predict the days of survival during drought for a given rosette size by calculating and plotting the 95% prediction interval assuming simple linear regression (Figure 6c). The days of survival during drought and their corresponding rosette size of the selected transgenic lines from Figure 6b were then overlaid on the 95% prediction intervals of Col‐0 (Figure S10). A few ABI3:*SAL1* and LEC1:*SAL1* individual transgenic plants showed improved drought tolerance relative to their counterparts with similar rosette sizes, including one of the ABI3:*SAL1* line #2 individuals (highlighted in Figure 6c).

## 4 DISCUSSION

A by‐product of secondary sulfur metabolism, PAP, was identified as a stress‐induced chloroplast retrograde signal based on the study of the Arabidopsis *sal1* null mutant (Estavillo et al., 2011). To further understand how PAP functions in interorganellar communication in plants to regulate stress responses and other physiological functions, it is crucial to identify multiple ways to manipulate and fine‐tune PAP levels. Nevertheless, no systematic investigations on such manipulation in Arabidopsis have been reported thus far. Biochemical manipulation by directly feeding the plants with the PAP chemical itself (Pornsiriwong et al., 2017) is possible but expensive and not amenable to long‐term manipulations. There have also been reports of expression of SAL1 isoforms, which either completely or partially complement *sal1* (Estavillo et al., 2011; Kim & von Arnim, 2009; Rodríguez et al., 2010), but these strategies still have limited resolution in “gradients” of PAP levels and do not allow specific temporal control of PAP accumulation. Therefore, we utilized and tested different established genetic manipulation tools such as hpRNAi and amiRNA in wild‐type Arabidopsis, as well as chemical‐inducible and developmental stage‐specific promoter for genetic complementation of *sal1*, to manipulate *SAL1* gene expression for PAP accumulation.

In our dex‐inducible SAL1‐silencing experiments using the pOpOff system (Figure 1), dex treatments could not be directly translated into PAP accumulation in Arabidopsis despite the slight reduction in SAL1 protein levels (Figure S1d). This lack of correlation is also supported by the absence of *sal1*‐like rosette morphology in all the transgenics after the prolonged dex treatment. These results cannot be explained by dex dosage or insensitivity to the dex treatment because strong GUS staining at targeted regions with dex painting (Figure S1a) confirms the high specificity and sensitivity of the dex‐inducible system and implying the expression of the *SAL1*hpRNAi construct as they are under the control of the same bidirectional promoter. Indeed, a gradient of GUS expression and *SAL1*‐silencing efficiency were detected in different transgenic lines as expected (Figure 2a,b). However, the extent of *SAL1* transcriptional repression improved from a maximum of 60% in the dex‐inducible hpRNAi lines to more than 80% relative to wild type when the same hpRNAi was driven by a strong promoter (Figure 2b), suggesting that the less efficient *SAL1* silencing in the dex‐inducible hpRNAi lines could be partially attributed to promoter strength.

Despite the stronger silencing of *SAL1* in the 35S:*SAL1*hpRNAi lines, PAP levels in these transgenic lines generally remained comparable to that of wild type at T1 and T2 generations (Figure 2c; Figure S3b). While there were some lines with seemingly higher‐than‐wild‐type PAP levels in Figure 2c, those levels were not reproducible in the subsequent generation and more importantly, there was no *sal1*‐like rosette morphology observed in any of these transgenic lines (Figure S3a). Hence, the “slightly elevated” PAP levels more likely reflect biological variation in PAP levels, or that these leaves were slightly stressed. The inability to induce PAP accumulation by silencing *SAL1* is unlikely to be due to the vector, as the pAgrikola vector used herein has been successfully utilized previously to constitutively repress gene expression for at least three other genes (Hilson et al., 2004). It is also unlikely to be due to the design of the SAL1 hpRNAi construct, as similar results were obtained when two different 2X35S:*SAL1*amiRNA constructs were tested. Again, *SAL1* transcript levels were reduced to around 25% of that of wild type in transgenic lines (Figure 3b), but no PAP accumulation can be detected in any of them (Figure 3c,d). It is interesting that despite changing silencing methods and targeting regions within the *SAL1* transcript, no increment in PAP levels was detected and a maximal *SAL1* transcript reduction of ~80% relative to wild type was maintained (Figure S5). This suggests that failure of the *SAL1‐*silencing strategies to induce *sal1* phenotypes is influenced by biological, not technical, factors.

What are the possible explanations for our observed lack of correlation between *SAL1‐*silencing efficiencies and PAP accumulation? One possibility is that silencing of *SAL1* did initially cause PAP accumulation, but then PAP could suppress *SAL1* silencing due to its role in RNA metabolism (Gy et al., 2007), thereby creating a feedback loop that restores PAP levels to WT. However, this would have led to high *SAL1* transcript levels across most of the transgenic lines and yet we observed good silencing efficiencies of up to 80% even in *35S* lines. Additionally, we performed 35S:*PDS* hpRNAi to silence carotenoid biosynthesis in *sal1* and still observed the expected bleaching phenotype (Figure S6), suggesting that gene silencing can still proceed to an adequate level when PAP accumulates.

Another interpretation of the inability to induce PAP levels despite a massive reduction in *SAL1* transcript levels is that SAL1 protein is a relatively stable protein with very low turnover rate or that reduction in the SAL1 protein levels achieved in the transgenic lines is insufficient to affect SAL1 catabolic activity against the low level of PAP in vivo. The kinetic parameters of Arabidopsis SAL1 (Chan, Mabbitt et al., 2016) indeed suggest that SAL1 is a very efficient protein when compared to other secondary metabolism enzymes (Bar‐Even et al., 2011). Enzyme that functions in secondary metabolism typically “operates under specific conditions or for short periods of time and at relatively low fluxes” (Bar‐Even et al., 2011). It is likely that SAL1 in wild type is normally present in excess compared to the comparatively low metabolic flux into PAP production during normal plant growth. Consequently, the reduced levels of SAL1 present in the transgenic lines (Figure S1d) together with other reported PAP catabolic enzymes such as AHL (Hirsch et al., 2011) could be sufficient to maintain the wild‐type‐like PAP levels under standard growth conditions.

The same factors, promoter strength and SAL1 activity, which limited the effectiveness of the four different silencing strategies, could have underpinned the success of temporal‐specific SAL1 expression in *sal1*‐6. With varying dex treatments during plant growth, PAP levels and rosette growth of pOpON‐*SAL1* transgenics were successfully altered (Figure 4); where prolonged dex treatment during plant growth results in lower PAP levels (Figure 4d) and faster rosette growth (Figure 4b,c). Interestingly, the presence or absence of SAL1 during seedling development (i.e., first 2 weeks of growth on plates since germination) did not yield significant differences in promoting rosette growth relative to the empty vector control, whereas only 1 week of dex treatment for SAL1 induction during vegetative growth stage is sufficient to marginally improve rosette growth. This suggests that low PAP levels during rosette development are critical to ensure proper rosette expansion in Arabidopsis. Whether this is linked to the reported influence of PAP on auxin (Robles et al., 2010; Zhang et al., 2011), ABA (Pornsiriwong et al., 2017; Rossel et al., 2006) or jasmonic acids (Rodríguez et al., 2010), or the interaction between SAL1/PAP and light perception (Chen & Xiong, 2011; Kim & von Arnim, 2009) will need to be further investigated, given that both phytohormones and phytochromes are known to regulate rosette growth and development.

Interestingly, despite successful repression of PAP levels at the end of the dex treatment regime, none of the pOpON:*SAL1* transgenic lines appear to have fully reverted rosette phenotypes. In contrast, when we utilized promoters of genes (*ABI3*,* TZF6*, and *LEC1*) known to express specifically at early developmental stages (during embryogenesis up to germination stage) to drive the complementation of *sal1*, plants with fully complemented rosette phenotypes were obtained, but none of these possess high PAP levels after 4 weeks of growth (Figure S8). Our observation thus far likely suggests that only *ABI3* promoter showed developmental stage‐specific expression. The apparent non‐seed‐specific expression of *TZF6* in particular could suggest that the full proportion of *TZF6* promoter was not captured in this experiment. Alternatively, *TZF6* expression is known to be induced by high ABA and jasmonic acid levels (Bogamuwa & Jang, 2013), which are also reported effects of PAP accumulation in the absence of functional SAL1 (Estavillo et al., 2011; Rodríguez et al., 2010; Rossel et al., 2006). Nonetheless, when taken together, these observations suggest that low PAP levels throughout rosette development are necessary for developing wild‐type‐like leaf shape and rosette size and that high PAP accumulation at certain stages of plant development could lead to irreversible effects on morphology. Future detailed analysis using the transgenic lines generated herein could allow dissection of the role of SAL1‐PAP in regulating rosette growth and leaf development, for instance by systematically tracking the *SAL1* transcript, protein and PAP levels in relation to other major regulators of leaf morphology during plant development.

Significantly, a number of lines from the dex‐inducible and developmental stage‐specific complementation strategies retained comparable drought tolerance compared to the empty vector and no promoter negative control, respectively, despite having a larger rosette size, and/or better drought tolerance than the Col‐0 control despite having comparable rosette sizes (Figures 5 and 6c). Whether or not these favorable traits are stable and heritable to subsequent generations and if the tradeoff between rosette growth and drought tolerance by balancing the PAP accumulation in Arabidopsis can be defined or further refined will require more meticulous characterization in the future. Nevertheless, our results herein are consistent with our previous work showing that the drought tolerance of *sal1* can be independent of rosette size (Wilson et al., 2009). Therefore, the heritability of the optimal balance between rosette growth and drought tolerance via SAL1‐PAP manipulation could be of biotechnological interest (at least as a proof of concept). Our work herein has pinpointed potential genetic manipulation strategies and generated biological tools that can assist in refining in vivo PAP levels to study, and manipulate, its dosage effects on plant growth and drought tolerance.

## AUTHOR CONTRIBUTIONS

SYP performed all experiments in main figures; WP designed and/or cloned *SAL1*amiRNA and pOpOff‐*SAL1*hpRNAi constructs and performed initial characterization of the pOpOff‐*SAL1*hpRNAi transformants; SYP, KXC, GME, and BJP led planning, analysis, and manuscript preparation.
