# Supplementary material for: Development of strategies for genetic manipulation and fine‐tuning of a chloroplast retrograde signal 3′‐phosphoadenosine 5′‐phosphate
Source: Plant Direct. 2018 Jan 9;2(1):e00031. doi: 10.1002/pld3.31 (PMC6508504; doi:10.1002/pld3.31)
Supplement: Supplementary file 1 [file PLD3-2-e00031-s001.pdf]

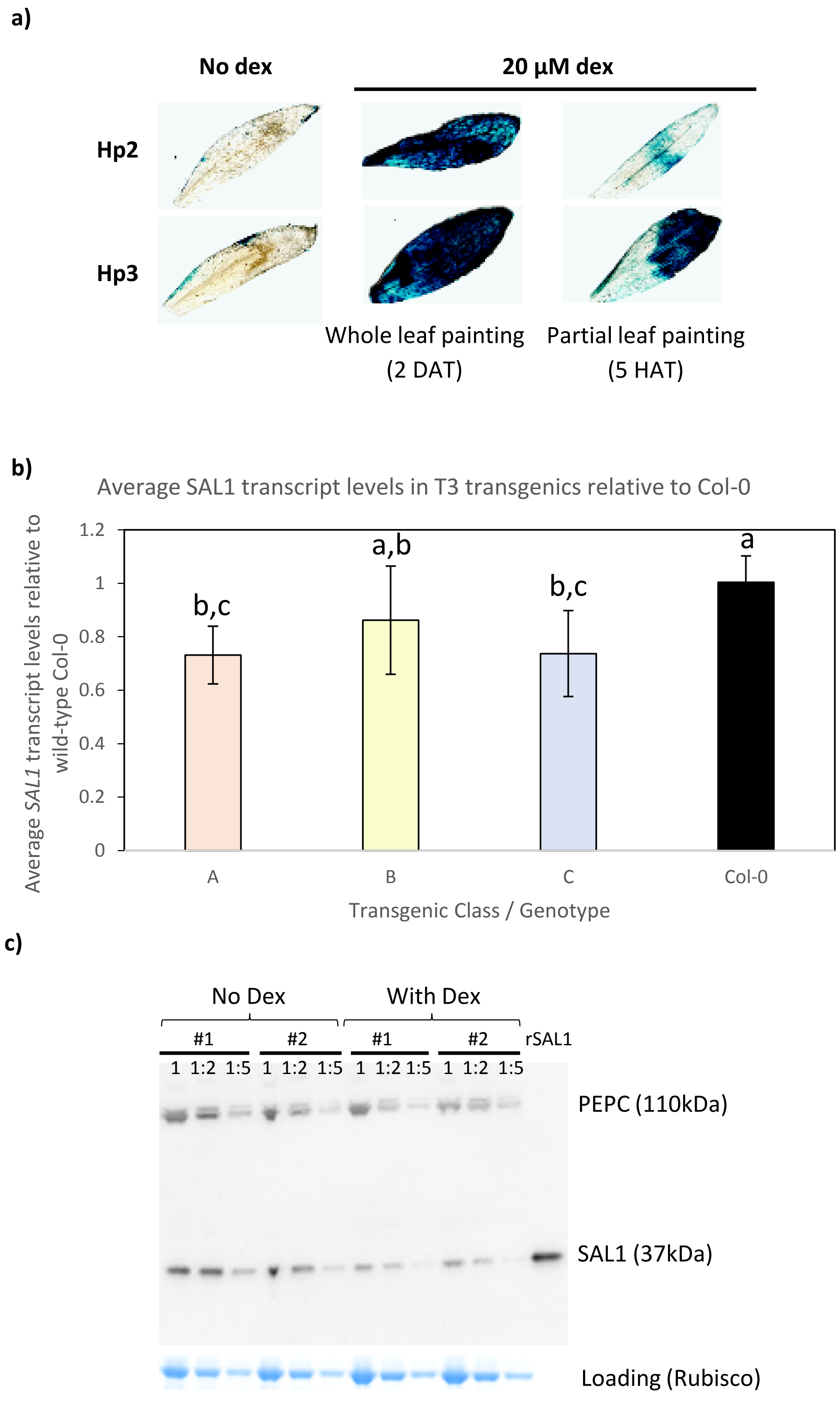

Supplementary Figure 1 – Preliminary testings of the dexamethasone-inducible system using a few selected pOpOff-*SAL1*hpRNAi lines in Arabidopsis. A) Leaves of T1 transformants were either painted with no dex or with 20 µM dex before GUS staining was checked 2 days after treatment (DAT) on whole leaf or 5 hours after treatment (HAT) on partial leaf painting. B) *SAL1* transcript quantification from leaf tissues of three-week-old T3 plants after one week of 20µM dex treatment via soil-drenching. n=3 and error bars indicate standard deviation. Significant differences (ANOVA, $p<0.05$) are denoted by a, b, c. C) Total protein was extracted from leaves of four-week-old T3 plants treated with or without dex via soil drenching every three days since germination (n=2), SAL1 and PEPC as loading control were probed. Dilution series of 1:1, 1:2 and 1:5 were loaded for each sample.

a)

Line #	GUS Staining Intensities (5 replicates)			Class
	Strong	Moderate	Weak	
8	5	0	0	A
12	5	0	0	A
17	5	0	0	A
19	5	0	0	A
21	5	0	0	A
36	5	0	0	A
10	4	1	0	B
11	4	1	0	B
22	4	1	0	B
38	4	1	0	B
9	3	1	1	C
13	3	1	1	C
16	3	1	1	C
23	3	1	1	C
25	3	2	0	C
33	3	1	1	C
28	2	0	3	D
34	2	3	0	D
35	2	3	0	D
29	0	2	3	E
37	0	0	0	False Positive

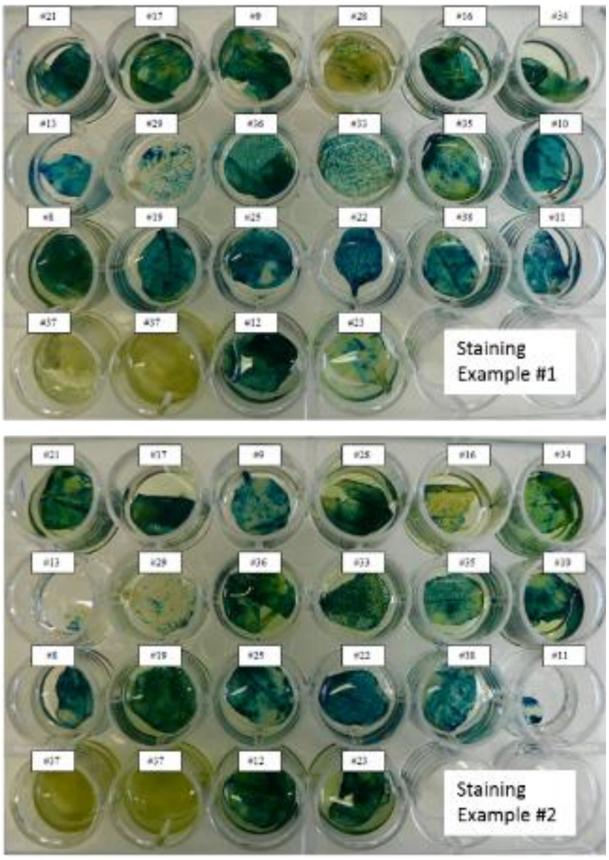

b)

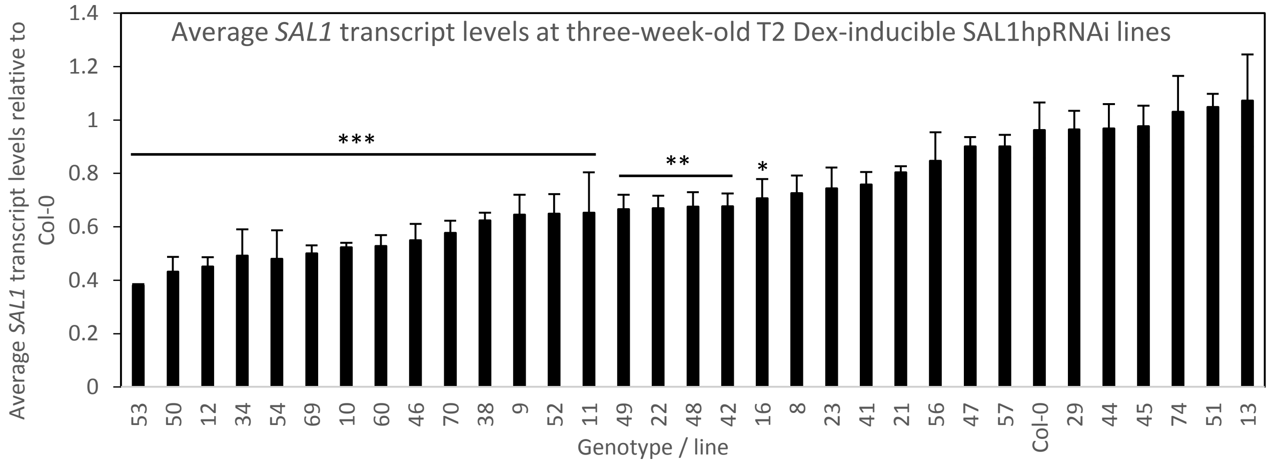

c)

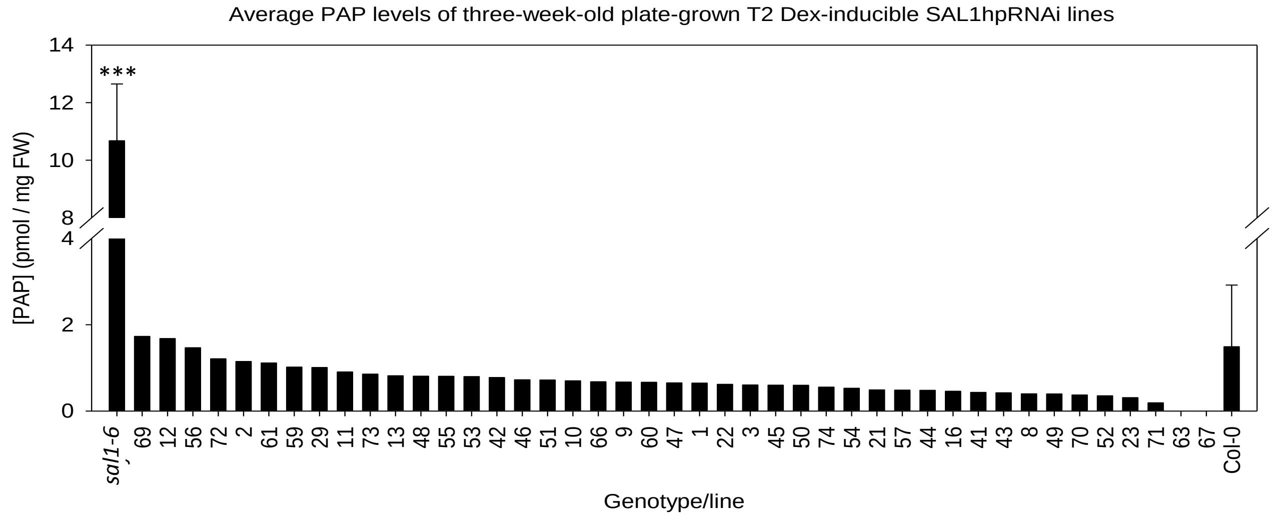

Supplementary Figure 2 – Screening transgenic lines to identify the potential of *SAL1* silencing efficiency of pOpOff system. A) Leaves of four-week-old T2 plants were harvested for GUS staining two days after painted with 20µM dex. Three-week-old seedlings germinated and grown on MS supplemented with 20µM dex were harvested for B) *SAL1* transcript quantification relative to the wild-type Col-0 control via qRT-PCR [n= 3 technical replicates, error bars = standard deviation, significant differences = ANOVA, post-hoc test relative to Col-0: *** denotes p<0.001, ** denotes p<0.01 and * denotes p<0.05] and C) PAP quantification using HPLC. At least 10 seedlings per transgenic line were pooled for each quantification [n=3 biological replicates for Col-0 and *sal1-6,* error bars = standard deviation]. Student T-Test relative to Col-0 control was performed; *** denotes p-value<0.001.

a)

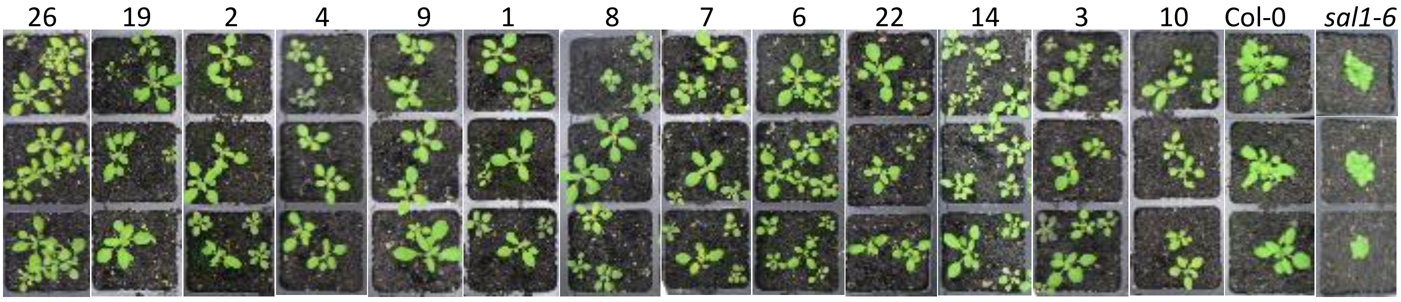

b)

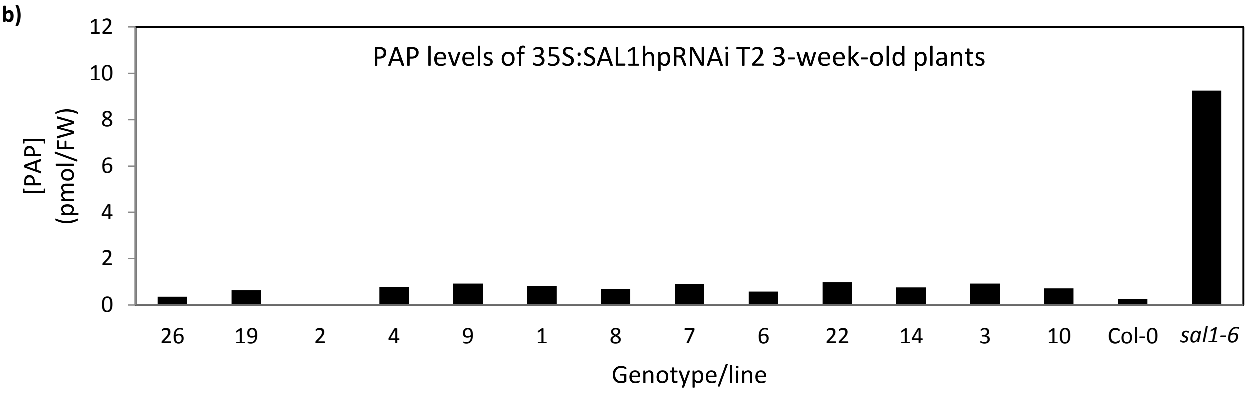

Supplementary Figure 3 – Characterization of T2 35S:*SAL1*hpRNAi lines. A) Representative photos of selected lines at 3.5 weeks old are shown. B) Selected 35S:*SAL1*hpRNAi lines, particularly those with "higher-than-Col-0" PAP accumulation at T1 generation, were re-tested for PAP levels at T2 generation, where five seedlings per line were pooled.

a)

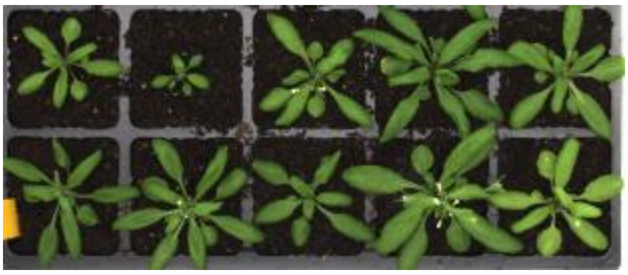

b)

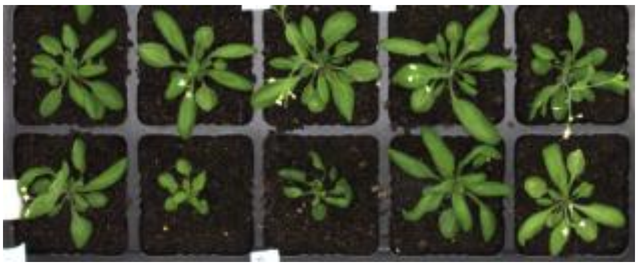

Supplementary Figure 4 – Representative images of T1 35S:*SAL1*amiRNA lines at four weeks old. A) 35S:*SAL1*ami339. B) 35S:*SAL1*ami1002. Each pot represents one independent transgenic line.

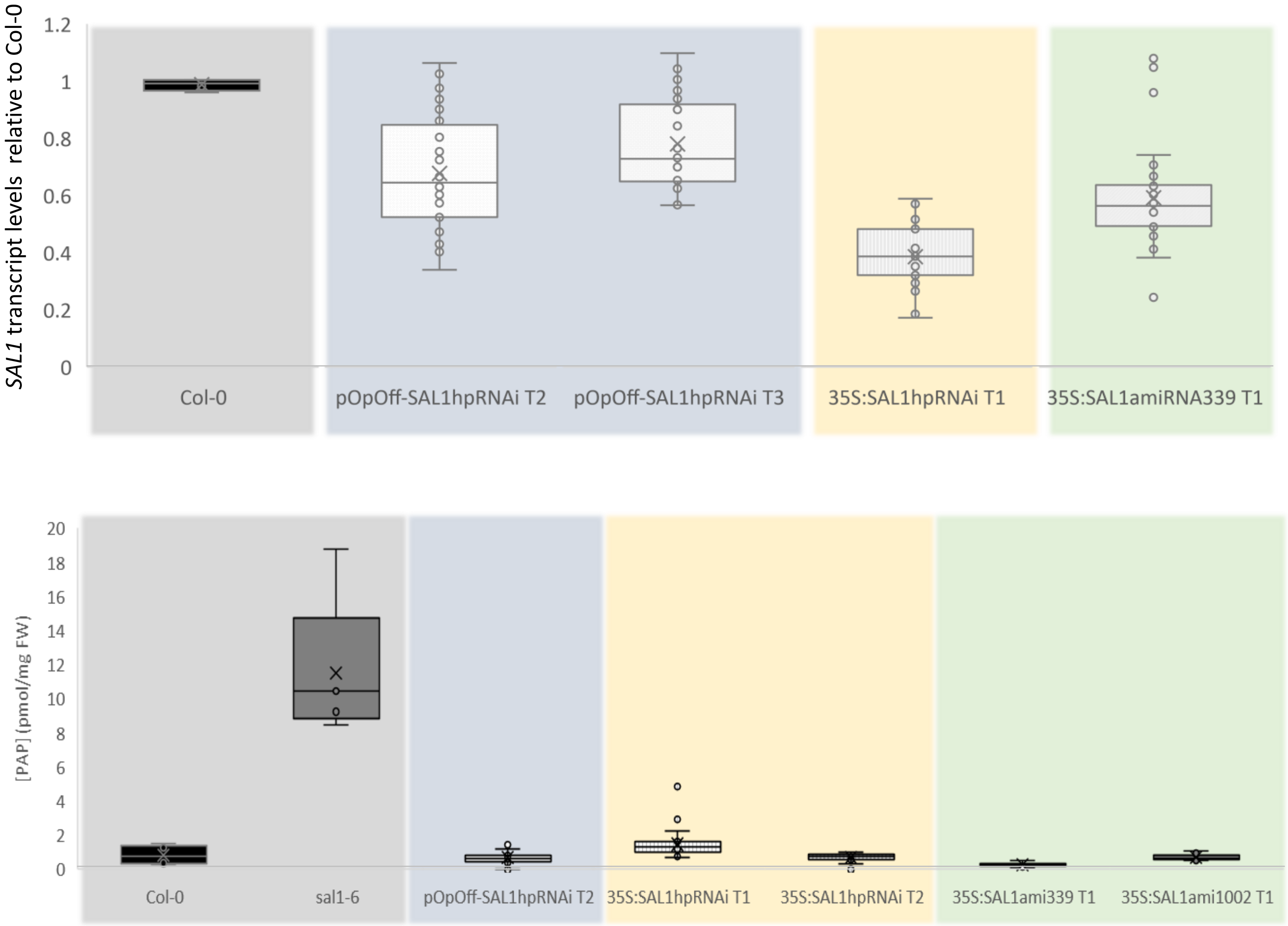

Supplementary Figure 5 – Summary findings of *SAL1* silencing strategies using: dex-inducible *SAL1*hpRNAi (blue), strong constitutive hpRNAi (yellow) and amiRNA (green). A) Boxplot of SAL1 transcript levels relative to wild-type Col-0. B) Boxplot of PAP levels.

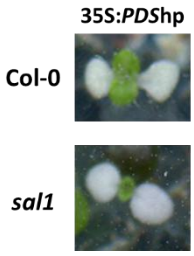

Supplementary Figure 6 – **Silencing of *PDS* in Col-0 and *sal1* transformed with 35S:*PDS*hpRNAi.**

Vector	Empty Vector						pOpON*SAL1*					
Dex treatment on germination	No			Yes			No			Yes		
Dex treatment duration on soil (weeks)	0	1	2	0	1	2	0	1	2	0	1	2
Abbreviation	EN0	EN1	EN2	EY0	EY1	EY2	PN0	PN1	PN2	PY0	PY1	PY2

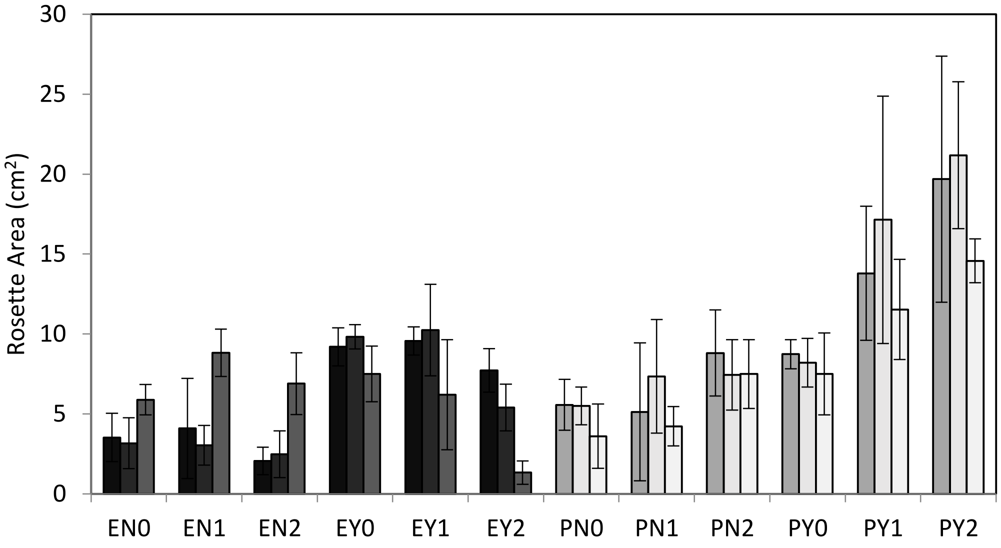

Supplementary Figure 7 – **Average rosette area of three independent transgenic lines of empty vector control and pOpON-*SAL1* at the end of the different dex treatment regimes.** Average rosette area of 3 to 4 plants per line at 38 days old was quantified using LemnaTec Scanalyzer and the error bars represent standard deviation. The abbreviated label on the x-axis is defined in the table above the bar graph.

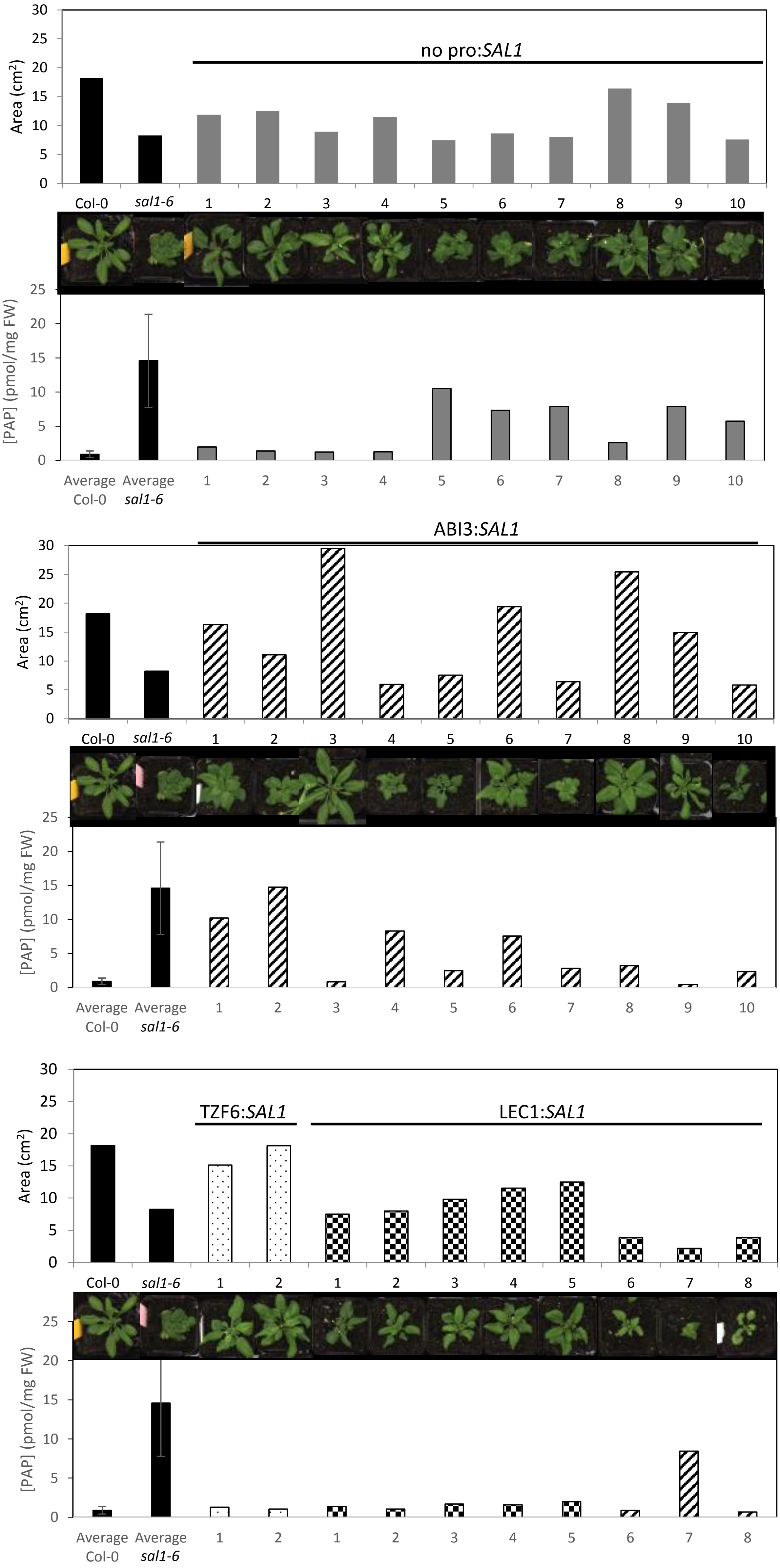

Supplementary Figure 8 – Rosette area, plant images and PAP quantification of individual T1 early developmental stage specific complementation of *sal1* transgenics together with Col-0 and *sal1*-6.

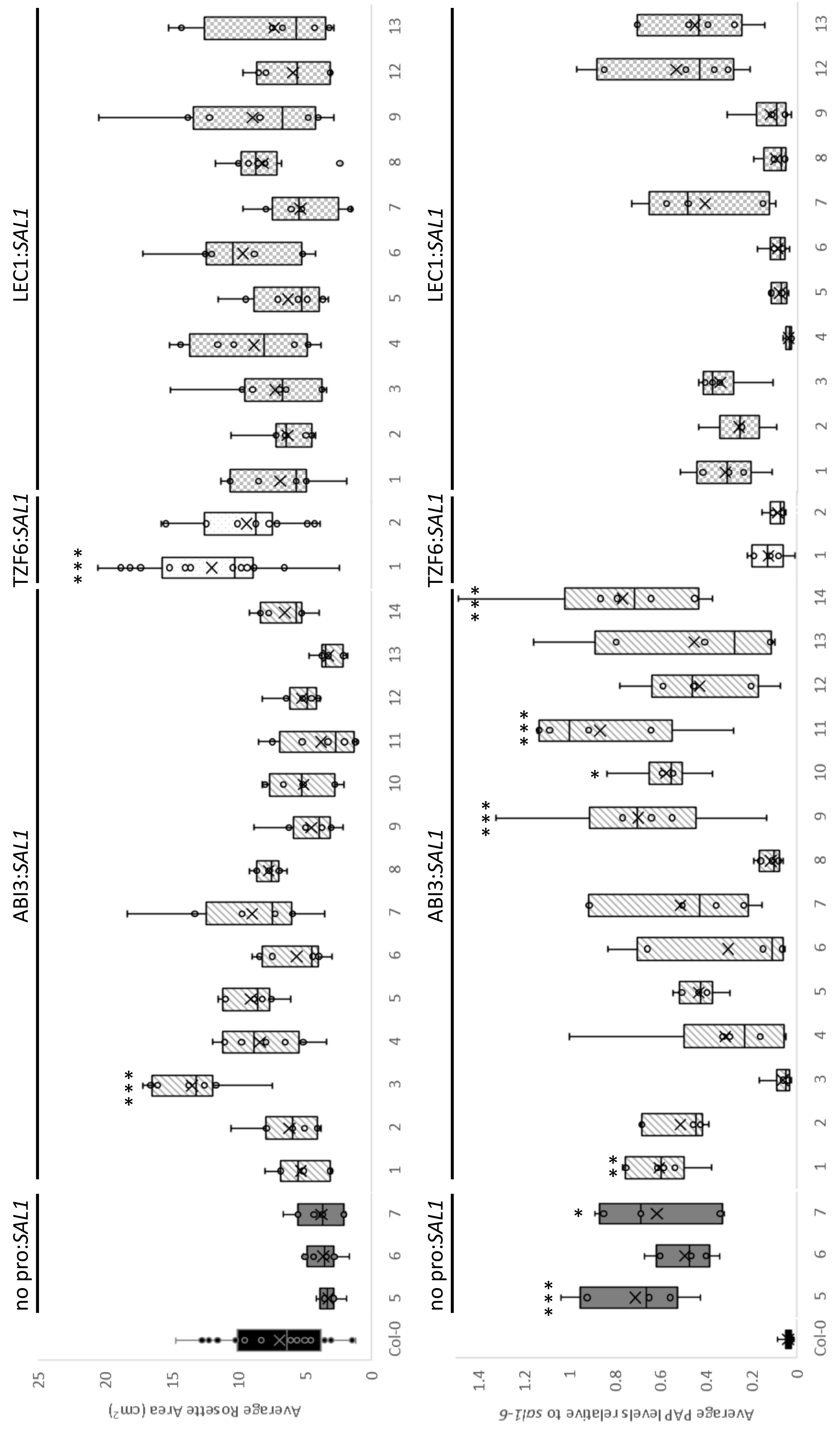

Supplementary Figure 9 – **Rosette area and PAP levels of T2 early developmental stage specific complementation of *sal1* transgenics in comparison to Col-0 and no pro:*SAL1* controls.** N≥5.

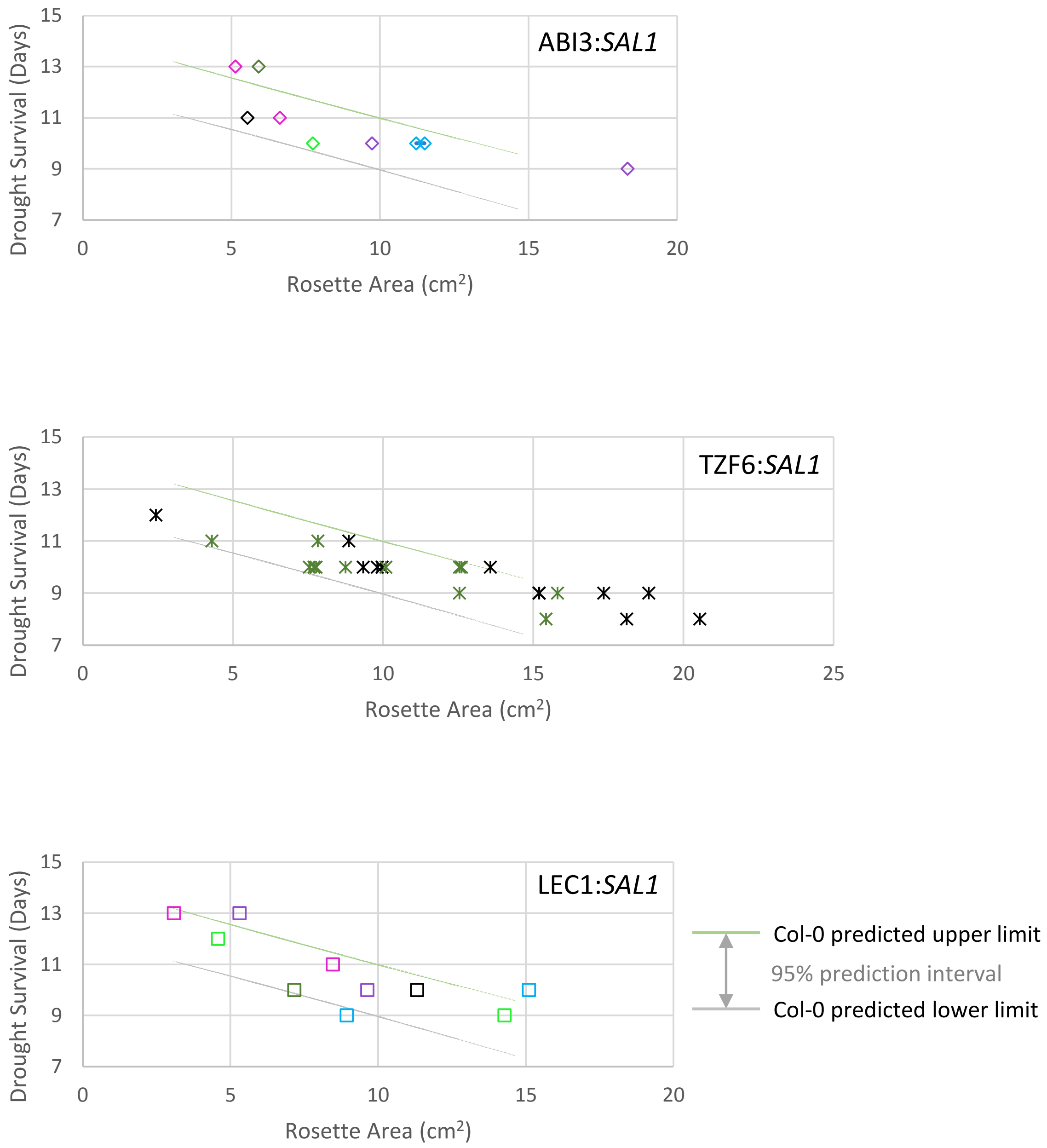

Supplementary Figure 10 – Days of survival during drought and their corresponding rosette area of T2 individuals of ABI3:*SAL1*, TZF6:*SAL1* and LEC1:*SAL1* plotted in comparison to the predicted upper and lower limits of Col-0 control. Different colours denote different independent lines.

```
   1 GACATATATT TATCTTCTTG AAAAGCGAAT GATGTCTATA AATTGTTTTC
  51 GAACAGCGAA GGCTCCGCTT CAATCATTTG TAGCAGTAAG AACGAATTCG
 101 AGACCTAGAA ATTCATCGAA CCGTCTCGTT TCTGTATTCG GACGCAAGTC
 151 TTCTTCTCCT TCATTTGTTA CTCTCAGAGT TGTTTCATCG ATGGCTTACG
 201 AGAAAGAGCT TGATGCTGCT AAGAAAGCTG CTTCACTCGC TGCTCGTCTC
 251 TGTCAGGTTA GGGTTTTTTC GATTCAATCA TGACCCATAG ATTCTAAAGT
 301 TTGATTCTTT AAGAAACCCA TTTTGTAAAT CTTCCAAATT TCGTTTAACA
 351 TTTTGTGTTT ATTGTGCATT GCATCTGTAA TTGGGAATAG ATTCTAGTGA
 401 TATAGTGTAA TGGTCCTCTA CATACGAAGC TCGTGTAAAT CTTTGATCAA
 451 AATCTTATCT TTGTGTTTTG GGTTTGTTTC AGAAAGTTCA AAAGGCTTTG
 501 TTGCAATCAG ATGTGCAATC AAAATCTGAT AAAAGTCCAG TGACCGTTGC
 551 TGATTATGGT TAGTTTGTTA TACCTGTCCC TGATTAGAAA AAGCTCTTCT
 601 CTTTGAATGT TACTGAGATT GTTAGGAAAT CACTTAATTT GATCTGTCTT
 651 GTGTTGAATT TCAGGTTCAC AAGCAGTTGT TAGTTTAGTC TTAGAAAAAG
 701 AGCTCAGTTC TGAACCCTTT TCATTGGTGG CTGAAGAGGT GAAACTGCTT
 751 AATAAATCCT TGTTAGATGT CTCACACTTT ACTTATCTTT GAGTTTGTGT
 801 TTATGGACTC ACATTGTCTA AAATGATCTA TATAGGACTC AGGCGATCTA
 851 CGCAAGGATG GTTCTCAGGA TACTCTGGAG CGCATCACAA AACTCGTGAA
 901 CGACACTTTG GCTACCGAGG AATCGTTTAA TGGCTCTACT TTGTCTACTG
 951 ATGATCTACT TAGAGCCATT GACTGTGGAA CATCTGAAGG TGGTCCAAAT
1001 GGTCGACACT GGGTCTTGGA TCCAATTGAT GGCACTAAAG GGTACGTTTT
1051 AAAACTAACT AGCCTAAAGT CAAATCTTCT TATTTCAGAG AAAATGTAAA
1101 TTTGATAGAA TGTTGAGTCA GATGTTATGT TCCTGACACT GAGCATTTTC
1151 ATGATTTTAG ATTTCTGAGG GGAGATCAAT ACGCAGTAGC ACTAGGATTG
1201 CTCGAGGAAG GGAAAGTAGT TTTAGGTGTG CTTGCTTGTC CAAACTTGCC
1251 GTTAGCATCC ATAGCAGGAA ACAACAAGAA CAAATCTTCG TCAGACGAAA
1301 TTGGATGCCT CTTCTTTGCT ACAATTGGTT CAGGGACATA TATGCAGCTC
1351 CTAGATTCAA AATCTTCTCC TGTAAAAGTG CAAGTCTCTA GTGTTGAGAA
1401 TCCTGAAGAG GCATCGTTCT TCGAGTCATT CGAAGGAGCT CACTCTCTAC
1451 ATGACTTATC CAGCTCCATT GCCAATGTAA ATTGCTTCTT TCCTTCCATG
1501 TGATTCCAGC TAATAGCTAA CTAATTTTCC TCATCCATTT GATCATGTTC
1551 TATGTTGTAA TATACAGAAA CTCGGTGTCA AAGCTCCACC AGTCCGTATT
1601 GATAGCCAAG CAAAGTATGG AGCTTTATCA AGAGGAGATG GAGCTATATA
1651 CTTACGGTTT CCTCATAAAG GATACCGCGA AAAGATTTGG GACCATGTCG
1701 CTGGTGCTAT AGTTGTTACA GGTAACATTA AAGCTTACTC TCTATGAAGC
1751 TAATTTTATA GTGTCGACAT GCGGATGTAA ATAGATAAGG AATGCAAGGT
1801 TGATTCTTCT TTTTGGTGCA GAGGCGGGTG GAATAGTGAC AGATGCAGCA
1851 GGAAAGCCAC TGGATTTCTC GAAAGGGAAG TATCTTGATT TGGACACAGG
1901 CATTATCGTT GCTAACGAGA AGCTAATGCC TCTGCTTTTG AAAGCAGTTC
1951 GTGACTCCAT AGCTGAGCAA GAGAAAGCTT CAGCTCTCTG ATTTGTTTTT
2001 TTCTCTCGTA CGTTCTTTGT TTCTCTGTAA CTGTTGTTTC ATTTTCTTTC
2051 ACCGAATTTC ACCAGTGAGA ATTTCTTCCA TTTTCGAAAA AGAAATAAAA
2101 ATGAAATTCT GTTTTGGGCT AA
```

amiRNA339 target region

Region targeted by SAL1hpRNAi

SAL1 qRT-PCR primers targeting region

amiRNA1002 target region

Supplementary Figure 11 - *SAL1* genomic sequence. Exons are highlighted in yellow. Region (excluding introns) indicated in black bracket is targeted by *SAL1*hpRNAi; blue box is targeted by 5'-targetting *SAL1*amiRNA; red box is targeted by 3'-targetting *SAL1*amiRNA; green bracket denotes region amplified during qRT-PCR for *SAL1* transcript quantification.

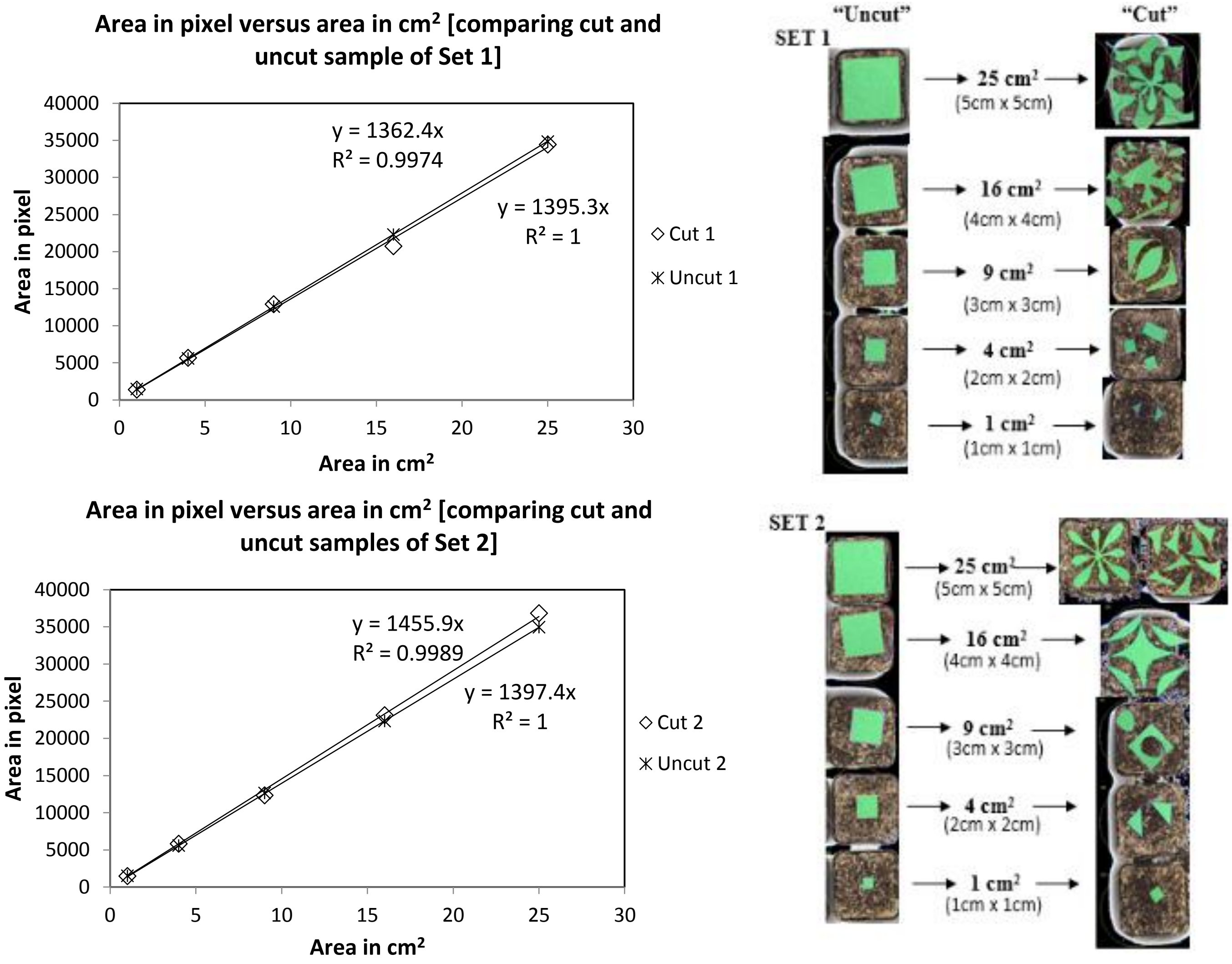

$$\text{Average conversion coefficient} = \frac{1362+1395+1455+1397}{4} = 1402.25$$

Supplementary Figure 12 – Calibration curve for the conversion of rosette area in pixel format from LemnaTec Scanlyzer software to area in cm². Green colour cards were cut into duplicated squares of specific dimensions (1cm X 1cm, 2cm X 2cm, up to 5cm X 5cm) and their images were captured and analysed using the Scanalyzer. These corresponding squares of known area were then cut into a series of interconnected pieces resembling an Arabidopsis rosette, or into disconnected pieces before images of them are captured and analysed again. This is to ensure the capacity of the image analysis to accurately detect and measure rosette area of Arabidopsis plants of varying size and rosette morphology.
