# Supplementary material for: Development of strategies for genetic manipulation and fine‐tuning of a chloroplast retrograde signal 3′‐phosphoadenosine 5′‐phosphate
Source: Plant Direct. 2018 Jan 9;2(1):e00031. doi: 10.1002/pld3.31 (PMC6508504; doi:10.1002/pld3.31)
Supplement: Supplementary file 2 [file PLD3-2-e00031-s002.pdf]

Reviewer Comments:
Reviewer #2:
Comments to the Author (Required):
The authors have addressed most of my previous comments. I would have preferred to have seen SAL1 transcript levels for the different transgenic lines produced, but the reference provided at least provides prior art of similar phenomena.

Reviewer #3:
Comments to the Author (Required):
In this study, the authors presented a precise regulation of PAP accumulation in chloroplast that balances stress tolerance and plant growth. I have read its previous version and comments from both reviewers, and also authors' response and revised manuscript. It seems all concerns have been well addressed. I don't have additional comments, and would recommend this manuscript to be accepted for publication.
